# Remodelling of P-bodies and the cytoskeleton by Orthohantavirus puumalaense (Puumala virus)

**DOI:** 10.1099/jgv.0.002220

**Published:** 2026-02-12

**Authors:** Hannah Sabeth Schwarzer-Sperber, Annette Petrich, Matthias Schade, Niklaas Nilson, Linah Chibrac-Ahad, Maik J. Lehmann, Katharina Paulick, Sabrina Weiss, Tina Dluzak, Daniel Bourquain, Peter T. Witkowski, Detlev H. Krüger, Andreas Herrmann, Roland Schwarzer

**Affiliations:** 1 Institute for the Research on HIV and AIDS-associated Diseases, University Hospital Essen, University Duisburg-Essen, Essen, 45147, Germany; 2 Physical Biochemistry, University Potsdam, 14476 Potsdam, Germany; 3 Institute for Biology, Humboldt University, 10115 Berlin, Germany; 4 Robert Koch Institute, Centre for Biological Threats and Special Pathogens, 13353 Berlin, Germany; 5 Technical University of Applied Sciences Bingen, 55411 Bingen, Germany; 6 Berliner Hochschule für Technik, 13353 Berlin, Germany; 7 Institute of Virology, Charité–University Medicine Berlin, 10117 Berlin, Germany; 8 Berlin Institute of Health at Charité, Universitätsmedizin Berlin, 10178 Berlin, Germany; 9 Free University Berlin, 14195 Berlin, Germany

**Keywords:** actin, cytoskeleton, fluorescence *in situ* hybridization (FISH), microscopy, orthohantaviruses, P-bodies

## Abstract

Orthohantaviruses are emerging zoonotic pathogens that can cause life-threatening diseases in humans. Their tripartite, negative-sense RNA genome is encapsidated by the viral nucleoprotein, but the subcellular localization and dynamics of these viral RNAs and proteins remain poorly characterized. Here, we present a comprehensive microscopy-based analysis of Puumala virus, the most prevalent orthohantavirus in northern and western Europe. Using fluorescence *in situ* hybridization (FISH) and Multiple Sequential FISH, we mapped the distribution of viral mRNAs, viral genomic RNAs (vRNAs), nucleoproteins and associated host cell factors, quantifying their intracellular abundance, co-localization and subcellular positioning. We observed distinct clustering of vRNAs with varying degrees of nucleoprotein association, a progressive increase in nucleoprotein expression levels during infection and a concomitant rise in the abundance of P-bodies. Moreover, we report a marked spatial reorganization of actin, microtubules and P-bodies, indicating substantial structural remodelling of host cells during orthohantavirus infections. Using a novel end-specific FISH assay, we observed a preferential 5′-end degradation of vRNAs in P-bodies, shedding new light on orthohantavirus RNA turnover within host RNA-processing compartments. Finally, co-localization analyses revealed the formation of potential ‘viral factories’ composed of nucleoprotein, vRNAs and viral mRNAs, indicating an intricate assembly hierarchy. Collectively, these findings improve our understanding of orthohantavirus replication and highlight the dynamic interplay between virus and host cell components.

## Introduction

Orthohantaviruses (family *Hantaviridae*) are a group of emerging, globally distributed viral pathogens that can cause serious and sometimes fatal diseases in humans [1]. Their proteome comprises only four components, which are encoded in the three negative-sensed, single viral genomic RNA (vRNA) segments designated S (small), M (medium) and L (large). The S-vRNA encodes for the viral nucleoprotein N, which encapsulates the viral genome in the virion. The fusogenic glycoproteins Gn and Gc are found on the M-vRNA segment as the glycoprotein precursor GPC. Finally, the L-vRNA encodes for the RNA-dependent RNA polymerase (RdRP). Notably, some orthohantavirus species, including Puumala virus (PUUV) [2], harbour an additional alternative reading frame in the S-vRNA that encodes a non-structural protein (NSs) with accessory functions [1].

Although the fundamental mechanisms and key processes underlying the orthohantavirus life cycle in the host cell have been comprehensively investigated, the exact site of the orthohantavirus replication has not yet been identified [3]. Cellular processing bodies (P-bodies), which are sites of RNA storage and degradation, have been reported to play a central role in the viral life cycle [3,5]. Furthermore, it has been suggested that orthohantavirus infections involve the formation of intracellular replication factories or viral factories [3,6], however clear evidence of such entities in orthohantavirus-infected cells is still lacking. Furthermore, the intracellular sites where the key replication processes occur and the spatiotemporal dynamics of viral molecules at those sites are hitherto unresolved. Therefore, many important questions regarding the complex crosstalk between different viral components, as well as between pathogen and host, remain unanswered.

In this study, we aimed to comprehensively decipher the interplay between orthohantaviral and host cellular factors. To this end, we investigated the localization and dynamics of all three orthohantavirus vRNA segments, viral mRNAs and N proteins using fluorescence microscopy. Additionally, we quantitatively examined different cellular factors – actin, tubulin and P-bodies – that play crucial roles in orthohantavirus entry, replication and assembly. Throughout the study, we focused on PUUV, a pathogenic Old-World hantavirus species (*Orthohantavirus puumalaense*), which is endemic to northern and western Europe, as a model Orthohantavirus species, for a thourough monitoring and analysis of intracellular infection events.

## Methods

### Cell culture and infection

If not otherwise stated, PUUV infection (strain Sotkamo: V-2969/81 Charite University Hospital, Berlin, Germany) was conducted in African green monkey kidney epithelial cells (Vero E6, ATCC CRL-1586; American Type Culture Collection, Manassas, VA) maintained in Dulbecco’s modified Eagle medium supplemented with 10% heat-inactivated FBS, 2 mM l-glutamine, 100 U ml^−1^ penicillin and 100 µg ml^−1^ streptomycin (all from PAA Laboratories GmbH, Austria) under standard cell culture conditions. Cells were passaged every 2–3 days when they reached nearly 80% confluence in tissue culture flask, for no more than 15 cycles. All infections were performed as previously described [7] and a brief outline can be found in the Supplementary Methods. Infection parameters [(m.o.i.=0.3–1; 24–240 h post-infection (hpi)] were chosen based on previously established conditions for orthohantavirus infection in Vero E6 cells, which provide robust replication kinetics suitable for microscopic and molecular analyses [7,9].

### Immunofluorescence and DAPI staining

Immunofluorescence and DAPI staining have been extensively described previously [7]. A detailed explanation can be found in the Supplementary Methods.

### Fluorescence *in situ* hybridization

Fluorescence *in situ* hybridization (FISH) probe design is described in the Supplementary Methods, and FISH probe sequences are listed in Supplementary Methods Tables SM1–SM3 (available in the online Supplementary Material). In a typical FISH experiment, samples were first treated with pre-warmed 80% formamide in 2× saline–sodium citrate buffer (SSC) at 37 °C for 10 min, to enhance FISH staining signals by increasing the accessibility of vRNA for probes. Subsequently, samples were rehydrated with 2× SSC buffer at room temperature (RT) for 10 min. Then, cells were incubated with hybridization buffer (200 nM FISH probes, 2× SSC, 10% formamide, 10% dextran sulphate and 2 mM vanadyl ribonucleoside complex) at 37 °C for 2–4 h, followed by two washing steps with pre-warmed 10% formamide in 2× SSC at 37 °C for 10 min. For FISH probe removal between sequential labelling steps [Multiple Sequential FISH (MuSeq-FISH)], an 80% formamide buffer wash was used, which decreases the melting temperature of double-stranded nucleic acids [10]. Of note, treatment with high concentrated formamide buffer impairs immunofluorescence (IF) staining, so all MuSeq-FISH experiments were conducted after antibody staining. In a typical MuSeq-FISH experiment, samples were imaged and then subjected to oligonucleotide probe removal by washes with pre-warmed 80% formamide at 37 °C for 10–15 min followed by rehydration with 2× SSC at RT for 5 min. Prior to the initiation of the new FISH cycle, successful removal of probes was verified by microscopy. Each FISH staining cycle labelled two different target RNAs and nuclei simultaneously. After completion of the final MuSeq-FISH cycle, cells were stained with HCS CellMask Deep Red (Thermo Fisher Scientific, Waltham, MA, USA) according to the manufacturer’s instructions and imaged together with DAPI to delineate cell boundaries for segmentation.

### Microscopy and image analysis

Stained samples were subjected to fluorescence microscopy, followed by manual or automated, quantitative image analysis. Details can be found in the supplementary methods.

### Creation of hierarchical assembly trees

A regression-based model for the linear relationship between the abundances of multi-segment complexes (MCC) of a certain complexity (rank *k*, *k ∊* [2,9]), and the products of the abundances of their constituent components with the corresponding rank *k*–1 was developed and thoroughly tested previously [10] (available via GIT-HUB (https://github.com/Budding-virus/Packbund). An example can be found in the Supplementary Methods.

### Statistical analysis

If not stated otherwise, bar charts show arithmetic mean±sem. Statistical significance was assessed using GraphPad Prism version 10 (GraphPad Software Inc., San Diego, CA, USA), applying parametric one-way ANOVA and Tukey’s multiple comparison tests and displayed as follows: ****P<0.0001, ***P<0.001, **P=0.001–0.01 and *P=0.01–0.05. Normality was tested using the Shapiro–Wilk test (α = 0.05).

## Results

In order to undertake a comprehensive investigation of orthohantavirus infections by FISH, probe sets were designed to detect all three genomic RNA segments as well as the corresponding viral mRNAs. As an initial step, we targeted PUUV vRNAs only to establish whether these viral nucleic acids and the viral N protein could be reliably identified and visualized within the same infected cell.

### FISH imaging identifies multiple types of PUUV vRNA/N clusters with distinct molecular signatures

**Fig. 1.**
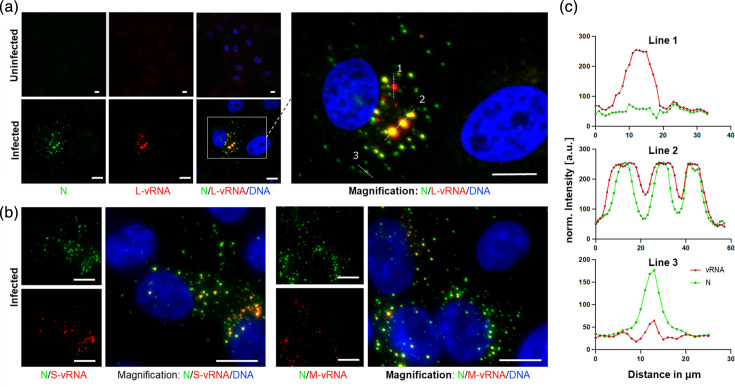
Concomitant imaging of different viral RNAs and N protein. Confocal images of Vero E6 cells subjected to IF staining and vRNA FISH at 72 hpi. (**a**) L-vRNA and N protein staining in uninfected (upper panel) and PUUV-infected cells (MOI=1, lower panel). Coloured labels indicate the displayed target molecule. The boxed area in the centre overlay image is shown magnified on the right. Thin dashed lines in the magnification image indicate regions of interest used for line plots in (c). (**b**) S-vRNA FISH and M-vRNA FISH in infected cells stained by IF for N protein. All images show maximum intensity projections of z-stacks, obtained by confocal microscopy. DAPI was used as a DNA counter staining (blue). Scale bars correspond to 10 µm. (**c**) Line plots, showing signals in the vRNA (red) and N protein (green) channels.

For a proof of concept, we performed FISH and IF at 72 hpi, a time point with robust steady-state infection levels [9]. Our experiment revealed virus-specific FISH signals for all three segments in infected cells, but not uninfected cells (Fig. 1a, b). Furthermore, we observed distinct populations of intracellular spots containing either vRNA, N protein or both (Fig. 1a, c).

### PUUV vRNA associates with actin accumulations, microtubules and P-bodies

**Fig. 2.**
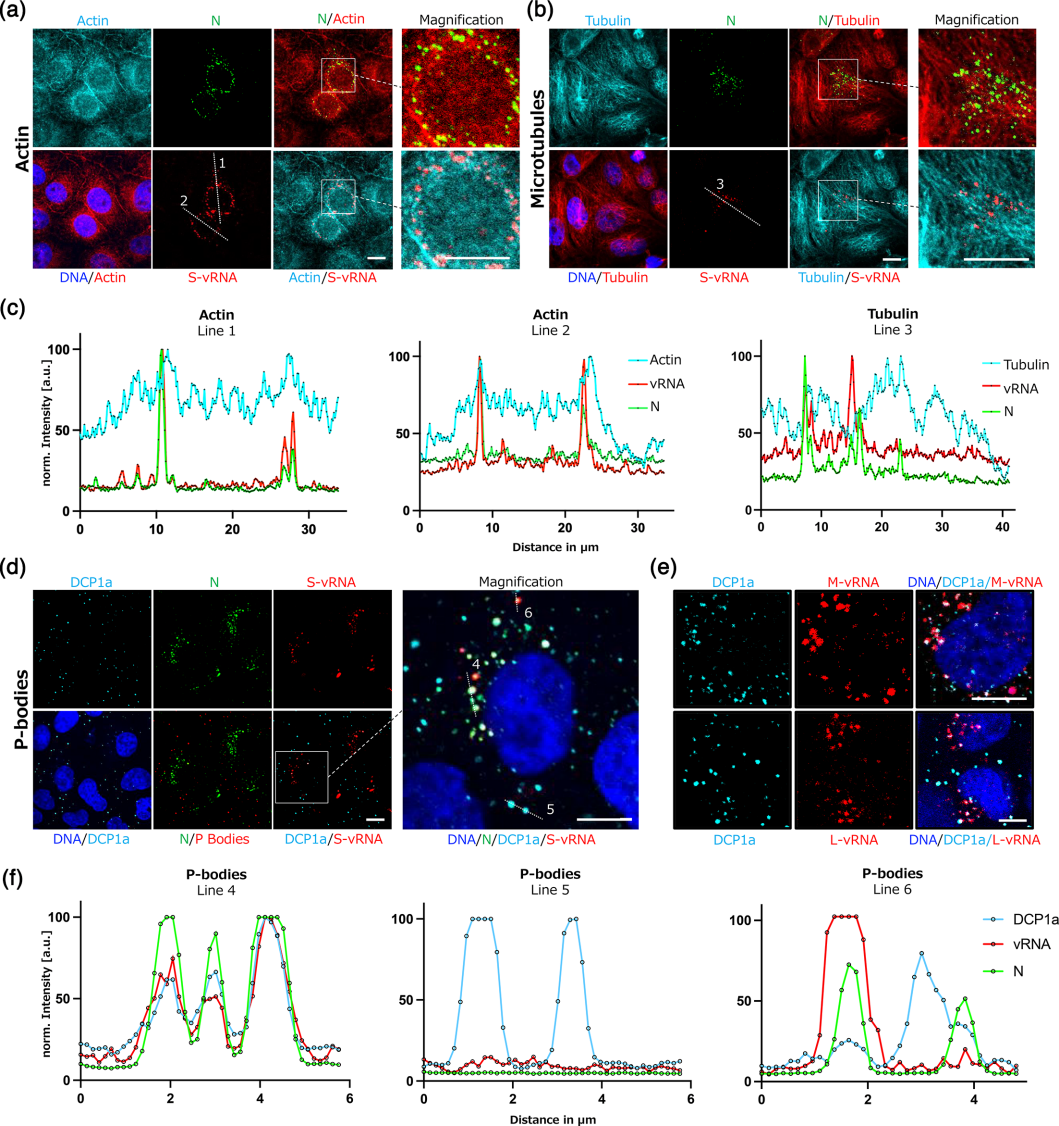
Combination of vRNA FISH and immunofluorescence staining for cellular factors. (**a**) Confocal image of a PUUV-infected Vero E6 cell (MOI=1, 72 hpi) stained for filamentous actin (TRITC-phalloidin, cyan), N protein (green), DNA (blue) and S-vRNA (red). (**b**) Confocal image of a PUUV-infected cell stained for *α*-tubulin (cyan), N protein (green), DNA (blue) and S-vRNA (red). The boxed regions are shown as magnifications. Scale bars=10 µm. (**c**) Line scan analysis showing normalized fluorescence intensity profiles for the dashed lines indicated in (**a**) and (**b**). (**d**) Confocal image of a PUUV-infected cell stained for P-bodies (DCP1a, cyan), N protein (green), DNA (blue) and S-vRNA (red). The boxed regions are shown as magnifications. Scale bars=10 µm. (**e**) Confocal image of a PUUV-infected cell stained for DCP1a (cyan), N protein (green), DNA (blue) and either M-mRNA or L-vRNA (red). (**f**) Line scan analysis of the dashed lines indicated in (**d**) and (**e**), showing normalized intensity profiles for DCP1a, N protein and the corresponding viral RNA marker. Additional example images of all three vRNA segments can be found in S1. Mock-infected cells are shown in Fig. S2.

Next, we assessed whether and to what extent vRNAs spatially correlate with different cellular factors. We focused on three key host components that we and others have previously been described to play important roles during orthohantavirus infection: actin, microtubules and P-bodies [4,7, 11,13]. Of note, our previous studies have demonstrated that infected cells exhibit a significant enrichment of filamentous actin in the perinuclear region when compared to mock-infected cells [7]. Our new findings now indicate that both the N protein and PUUV vRNAs co-localize with these actin accumulations (Figs 2a, c and S1). Similarly, N protein and vRNAs appeared to be lined along cellular microtubules (Figs 2b, c and S1). Furthermore, we detected a significant co-localization of all three vRNA segments with host P-bodies (Figs 2d–f and S1), which was often accompanied by N protein positivity in P-body/vRNA double-positive spots.

### PUUV infection induces redistributions of actin filaments, microtubules and P-bodies

**Fig. 3.**
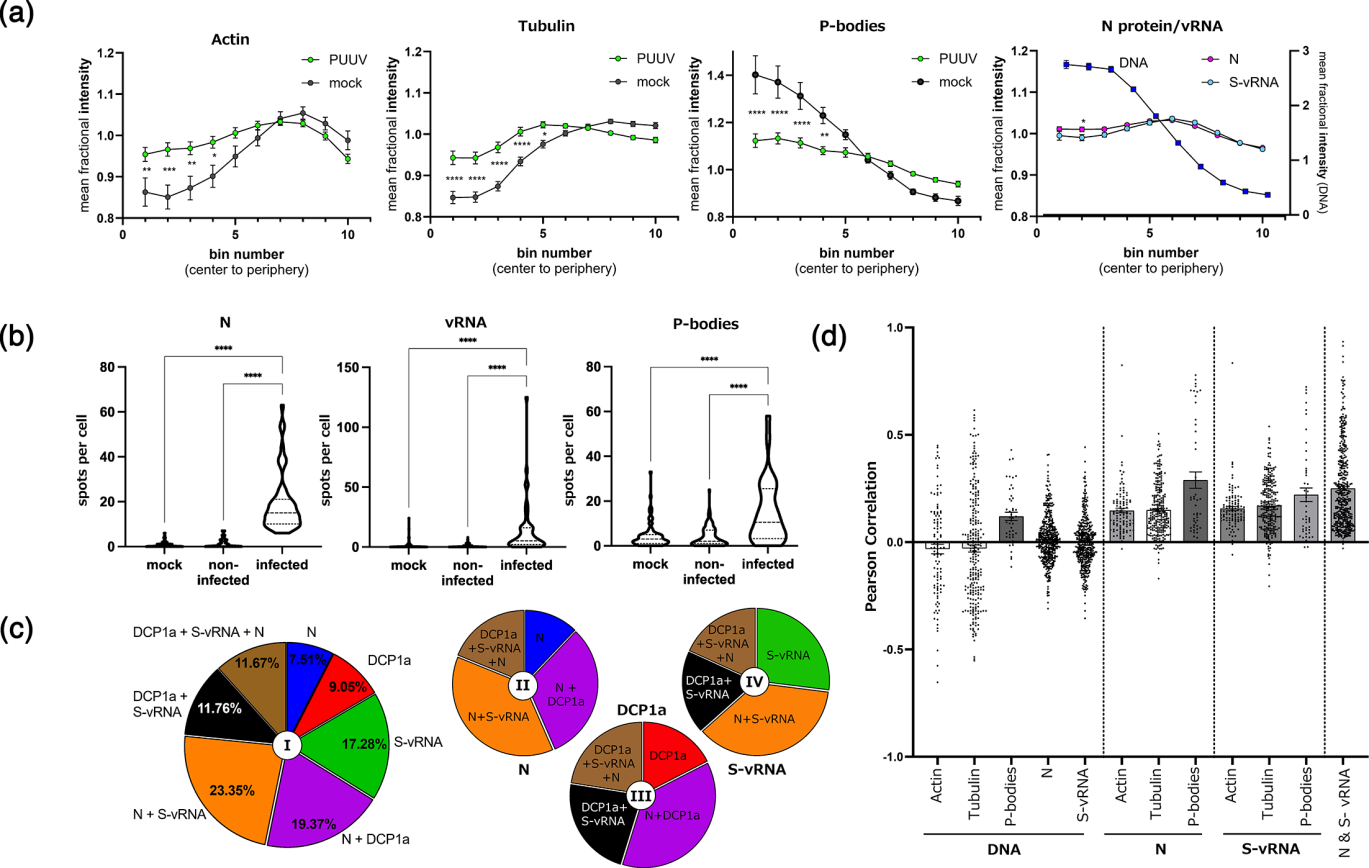
Quantitative image analysis of hantavirus-infected cells stained for vRNAs and cellular factors. (**a**) Automated image quantification was performed in CellProfiler. Segmented cells were divided into ten radial bins from the nuclear centre (bin 1, perinuclear) to the periphery (bin 10) using the MeasureObjectIntensityDistribution module. Then, MFrIs were calculated per bin. Mock-infected and PUUV-infected cells (*n*>30 per group) from multiple fields of view were included in the analysis. Error bars show the sem, and statistical significance was assessed by comparing PUUV and mock samples at each bin using a two-way ANOVA. The far-right plot indicates an analysis of N protein and S-vRNA intensities in infected cells. DNA MfrI is included as a control. (**b**) Numbers of identified N protein, P-body and S-vRNA spots in untreated (mock), virus exposed but uninfected (non-infected) and virus-infected (infected) cells, displayed in violin plots. Infected and uninfected cells were distinguished based on the N protein signal. Experiments were repeated three times (*n*=847 cells). ****P≤0.0001. Significance was analysed using two-way ANOVA and Tukey’s multiple comparison tests. (**c**) Relative co-localization of identified objects. Each section of the pie charts shows the percentage of a specific spot population with respect to all other spots in the respective analyses. The pie chart I summarizes all identified spot species (N, S-vRNA and DCP1a), whereas the smaller charts only focus on spots positive for N (II), DCP1a (III) or S-vRNA (IV), respectively. (**d**) Pearson correlation calculated per cell between indicated stainings. Individual cell measurements are shown as single dots (*n*>40), and all bars indicate mean with sem. Statistics are provided in Table S1.

To quantitatively assess changes in cellular structures induced by infection, we implemented automated image analysis using a custom CellProfiler pipeline applied to confocal microscopy datasets (Fig. S3). First, we sought to test whether PUUV infection alters the intracellular distribution patterns of actin filaments, microtubules and P-bodies. To that end, we calculated the mean fractional intensity (MFrI) of the respective proteins as a function of their distance to the cell centre and compared infected vs. mock-infected cells. Actin, *α*-tubulin and P-bodies were found to exhibit an aberrant intracellular distribution in infected cells (Fig. 3a), specifically in the centre and perinuclear region (bins 1–5). While actin and tubulin appeared to significantly accumulate in the cell centre, P-bodies redistributed from the inner to the outer regions of the cells (Fig. 3a). Expectedly, DNA was not displaced as a result of orthohantavirus infection (Fig. S4). For comparison, we also assessed the distribution of N protein and vRNAs in infected cells. N protein was chosen due to its crucial role and high abundance in orthohantavirus-infected cells. Accordingly, we focused on the S-vRNA, as this segment encodes for the viral N protein, enabling us to monitor the viral gene and its respective protein simultaneously. Notably, unlike the three cellular proteins investigated, viral RNA and the N protein showed significantly fewer divergent signals in the central vs. the peripheral regions of the cells (Fig. 3a, MFrI around 1), with hardly any statistical significance in their overall MFrI distribution.

### P-body numbers are increased in PUUV-infected cells

Next, we quantified the overall number of N proteins, DCP1a (P-bodies) and S-vRNA spots identified per cell for three different categories of cells: (1) PUUV-infected cells, (2) non-infected cells in PUUV-exposed cell cultures and (3) mock-infected (virus naïve) samples (Fig. 3b). In PUUV-exposed samples, cells were classified as infected or non-infected based on an N protein signal threshold defined in mock-infected samples. This naturally resulted in a strong significant difference in the number of N protein spots between mock or non-infected, and infected cells (Fig. 3b). Typically, between 10 and 20 N protein spots were identified in infected cells, whereas false-positive spots were rare (Fig. 3b). On average, ~13 S-vRNA spots were found in N protein-positive cells. Again, N protein-negative cells barely showed any false-positive spots. This indicates that our staining and analysis pipeline enables a reliable and consistent identification of different viral molecules in infected cells.

Interestingly, we observed a highly significant increase in the number of P-body spots in infected cells compared with non-infected cells and mock samples, which again points to a remodelling of cellular structures upon PUUV infection (Fig. 3b). Importantly, we also found a significant, albeit less extensive, redistribution and remodelling of both actin and P-bodies in a primary cell system based on human pulmonary microvascular endothelial cells (Fig. S5), indicating that this process is not cell-type specific. These quantitative results reinforce the idea that PUUV infection triggers significant remodelling of host RNA-processing compartments.

### Extensive co-localization of P-bodies with N and S-vRNA

To better understand the spatial relationship between host P-bodies and viral components, we then conducted a quantitative co-localization analysis. We found that almost half of all the spots identified (including those that were either S-vRNA, N or DCP1a positive) exhibited the P-body marker and at least one of the viral molecules (Fig. 3c, pie chart I: brown, magenta and black sections). Focusing on P-bodies, we found that the vast majority of all DCP1a-positive spots were associated with either N, S-vRNA or both (Fig. 3c, pie chart III). Similarly, the N protein and S-vRNA largely co-localized with P-bodies or each other, while only a small fraction was found not to be associated with any of the other investigated factors (Fig. 3c, pie charts II and IV, blue and green sections). Overall, these findings demonstrate a substantial spatial correlation between the N protein, S-vRNA and cellular P-bodies in PUUV-infected cells.

Lastly, we assessed the Pearson correlation of all stainings, by investigating the pixel-by-pixel co-localization of the target molecules under study (Fig. 3d). This analysis does not require identification of spots, nor are thresholds or other user-defined parameters necessary, making the results particularly un-biassed and objective. In line with our previously described findings, we observed the strongest positive correlations between N proteins and P-bodies, S-vRNA and P-bodies, as well as N protein and S-vRNA (Fig. 3d). A moderate positive correlation of N protein and S-vRNA was found with actin and *α*-tubulin, but also between DNA and P-bodies, likely indicating a nucleus-adjacent localization of P-bodies in PUUV-infected cells (Fig. 3d). As expected, no correlation was found between DNA and actin, *α*-tubulin, N protein or S-vRNA (Fig. 3d).

### Simultaneous imaging of vRNAs, mRNAs and N protein by MuSeq-FISH

The orthohantavirus life cycle involves three key molecular species: the genomic viral RNA (vRNA), its complementary positive-sense transcripts (mRNA and cRNA) and the encoded viral proteins [3]. Based on this rationale, we have designed a novel set of FISH probes that enable the parallel detection of vRNA, mRNA/cRNA and N protein. This covers the entire molecular replication sequence of the PUUV S segment (S-vRNA, S-mRNA and N). Notably, the FISH positive-sense probes used in this study hybridize to both mRNA and cRNA species. While cRNA serves as an essential replication intermediate [14], the most functionally relevant steps of the replication cycle, namely, genome amplification and viral protein synthesis, are governed by vRNA and the protein-encoding mRNA. Therefore, for simplicity purposes, the detected positive-sense RNA species are henceforward referred to as ‘mRNA’ throughout the manuscript.

**Fig. 4.**
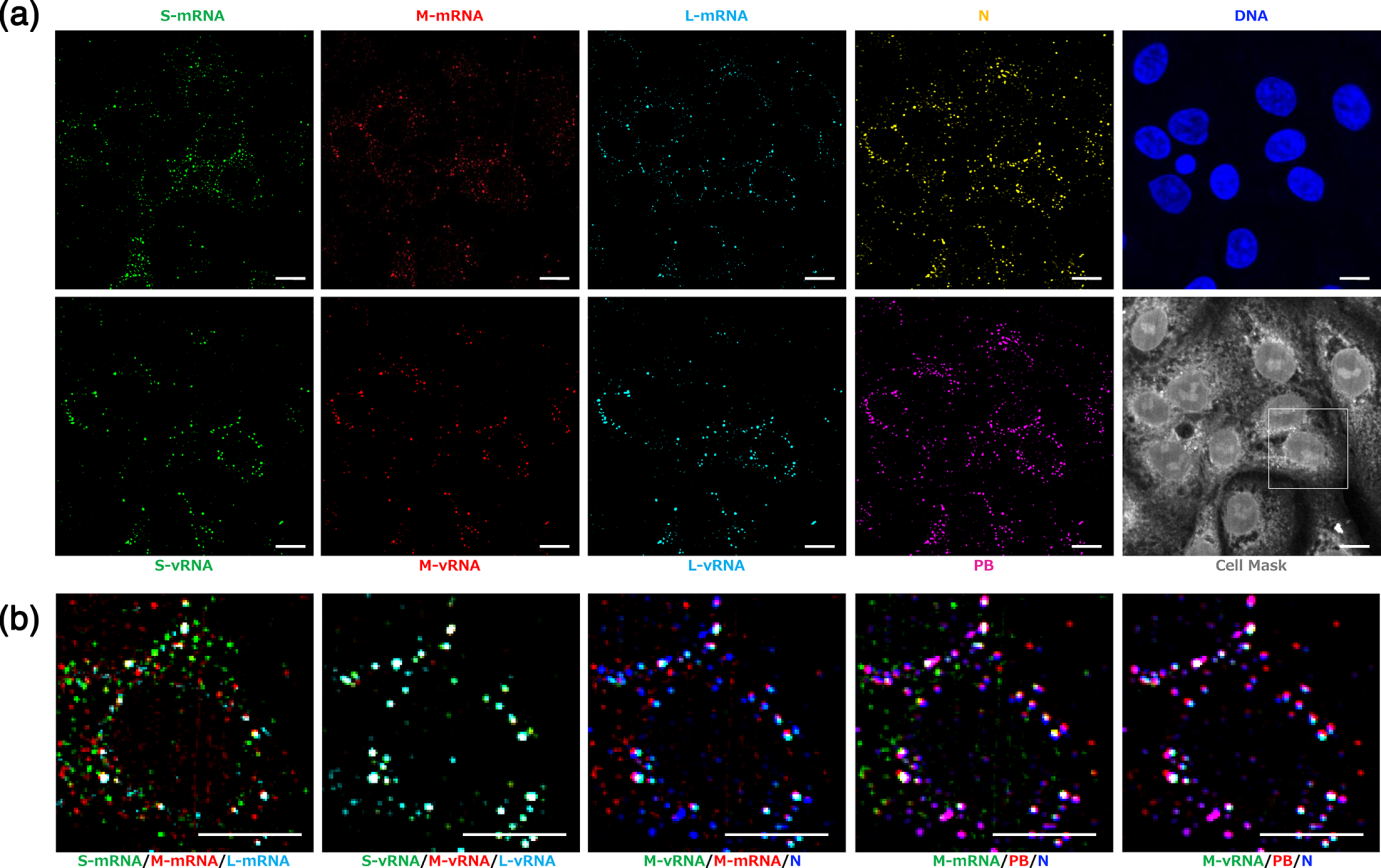
Utilization of MuSeq-FISH to image PUUV vRNAs and their transcripts in infected cells. Vero E6 cells were infected with PUUV (MOI=0.3) for 240 h and analysed using MuSeq-FISH, enabling consecutive imaging of multiple, viral nucleic acid species, viral and cellular proteins and DNA. (**a**) Overview images of a representative region of interest, showing each staining individually: S-mRNA and S-vRNA in green, M-mRNA and M-vRNA in red, L-mRNA and L-vRNA in cyan, N protein in yellow, P-bodies (PB) in purple, DNA in blue and CellMask staining in grey. (**b**) Magnification of the boxed region shown in the cell mask image in (**a**). Overlay images are shown as indicated below. Scale bars=10 µm. Images represent maximum intensity z projections. Additional images can be found in Fig. S7.

In order to be able to visualize all desired viral factors simultaneously, we employed a recently developed technique that enables the detection of numerous nucleic acid species in a single FISH experiment [10], called MuSeq-FISH (Fig. S6). We extensively applied this approach to stain PUUV-infected cells for all viral nucleic acid species (S-m/vRNA, M-m/vRNA, L-m/vRNA), as well as N protein and P-bodies over extended periods of time (up to 240 h). Representative micrographs at 240 hpi are shown in Figs 4 and S7. Importantly, we again observed spots with varying degrees of co-localization (Fig. 4a, b), including single stained puncta for each species, indicating the high specificity of all FISH probe sets utilized.

### MuSeq-FISH reveals continuous accumulation of viral mRNAs, vRNAs, N protein and P-bodies throughout the replication cycle

Subsequently, the comprehensive MuSeq-FISH datasets were investigated using FISH-Quant, which is a publicly available software for spot detection in 3D microscopy images [15], and a custom-written R script for localization and co-localization analysis [10]. In contrast to our previously used CellProfiler pipeline, this approach detects and measures vRNAs, mRNAs, N proteins and P-bodies in three dimensions. This improves and refines image segmentation and should result in a higher overall number of identified objects.

**Fig. 5.**
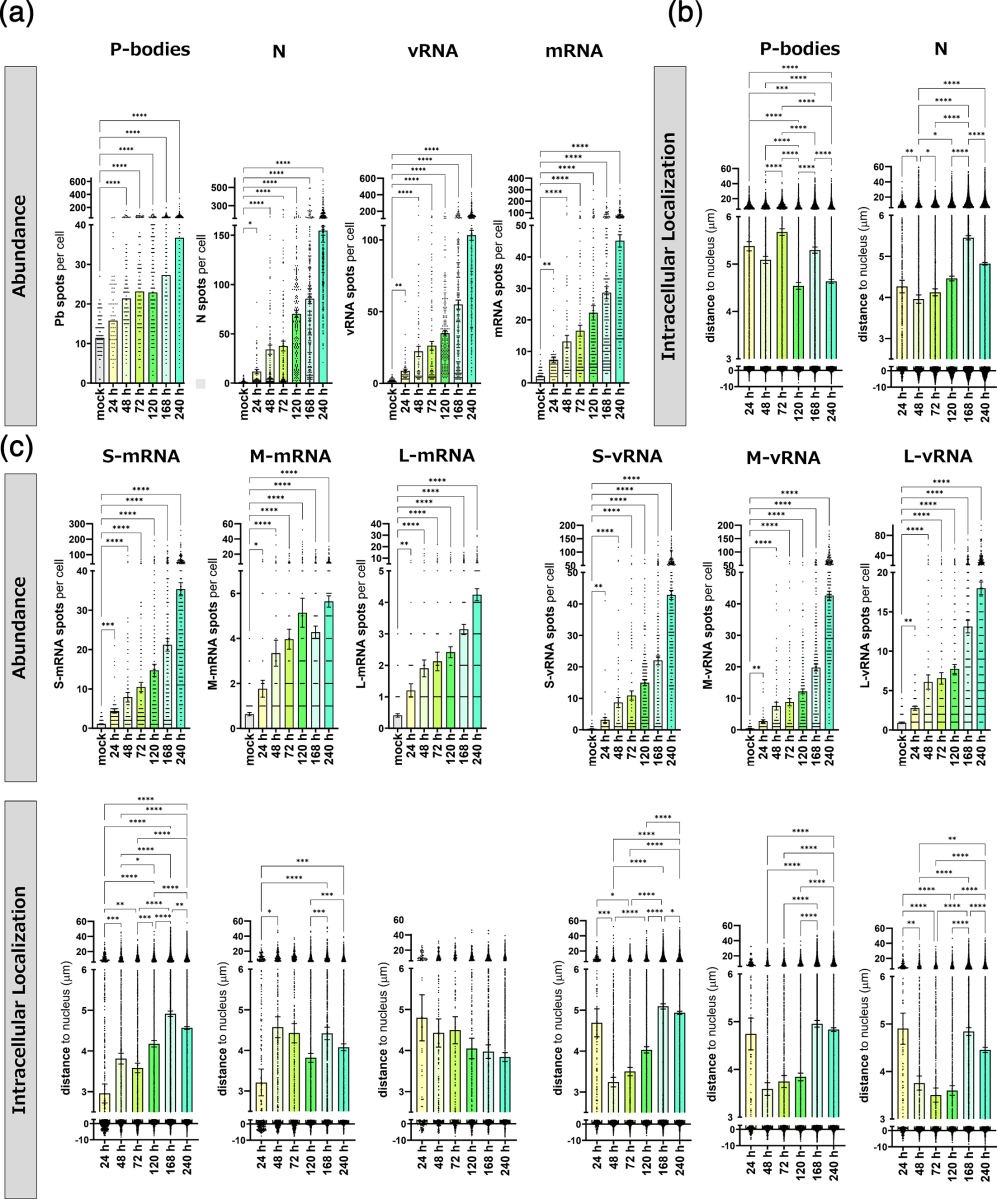
Quantitative analysis of PUUV infection kinetics obtained by MuSeq-FISH over 10 days of infection. Vero E6 cells were infected with PUUV and investigated at different time points post-infection using MuSeq-FISH as shown in Fig. 4. (**a**) Per cell counts (abundance) of P-body, N protein, all vRNA species and all viral mRNA spots in non-infected and infected cells. (**b**) Intracellular localization analysis, assessing the distance of each spot from the cellular centre of mass for P-bodies and N protein. Bars show the mean with sem and single cell values are displayed as dots for different time points post-infection. (**c**) Analysis of per cell counts and localization analysis for individual vRNA and viral mRNA species. ****P≤0.0001, ***P≤0.001, **P=0.001 to 0.01 and *P=0.01 to 0.05. Significance was analysed using one-way ANOVA and Tukey’s multiple comparison tests (*n*>50).

MuSeq-FISH revealed a progressive increase in the abundances of N protein, viral RNAs and P-bodies across all measured time points (24 to 240 hpi), highlighting ongoing viral replication and associated cellular responses (Fig. 5a). We also measured the distance of P-bodies and N protein puncta from the cell nucleus, finding significant changes at different time points (Fig. 5b). For P-bodies, the mean distance did not follow a clear trend, but N protein was found at increasing distances from the nucleus over time, with a maximum at 168 h. This could be indicative of heightened virus trafficking to the cell surface at late stages of the viral replication cycle. Next, we investigated individual vRNA and viral mRNA species, again assessing overall abundance and distance to the cell centre as a function of the infection time (Fig. 5c). As expected, all three vRNAs and their respective transcripts were found in gradually increasing numbers, as the infection spread and progressed in the cell culture (Fig. 5c, upper panel). Notably, the intracellular distribution diverged significantly across the different mRNA species and even more so between viral mRNA and vRNA (Fig. 5c, lower panel). Whereas S-mRNA and M-mRNA distance to the cell centre significantly increased over time, L-mRNA showed a trend towards shorter distances, albeit not significantly. In contrast, all vRNAs showed a high peak in distance at 24 hpi, followed by a significant drop at 48 hpi, with subsequent gradual increase at later infection time points (Fig. 5c, lower panel). We suspect the early peak to reflect the virus inoculum and thus be convoluted by virus entry, while the following gradual increase may indicate true peripheral egress events.

To validate our microscopy-based findings, we additionally conducted a strand-specific quantitive PCR (qPCR). For that purpose, we quantified vRNAs and viral mRNAs in cell pellets and cellular supernatants at various time points post-infection Fig. S8. Overall, the qPCR data were highly consistent, strongly supporting our microscopy results (Figs 5 and S8). For example, both methods showed a higher abundance of vRNAs than mRNAs, as well as higher levels of S-mRNA/vRNA than L-mRNA/vRNA (~factor 10). Moreover, our qPCR results indicate that mRNA levels plateau after 120 hpi, a finding that was also observed by FISH for M-mRNA. In strong contrast, however, both qPCR and FISH show that vRNA levels continue to rise beyond 120 hpi.

### N protein becomes the dominant factor at late infection stages

**Fig. 6.**
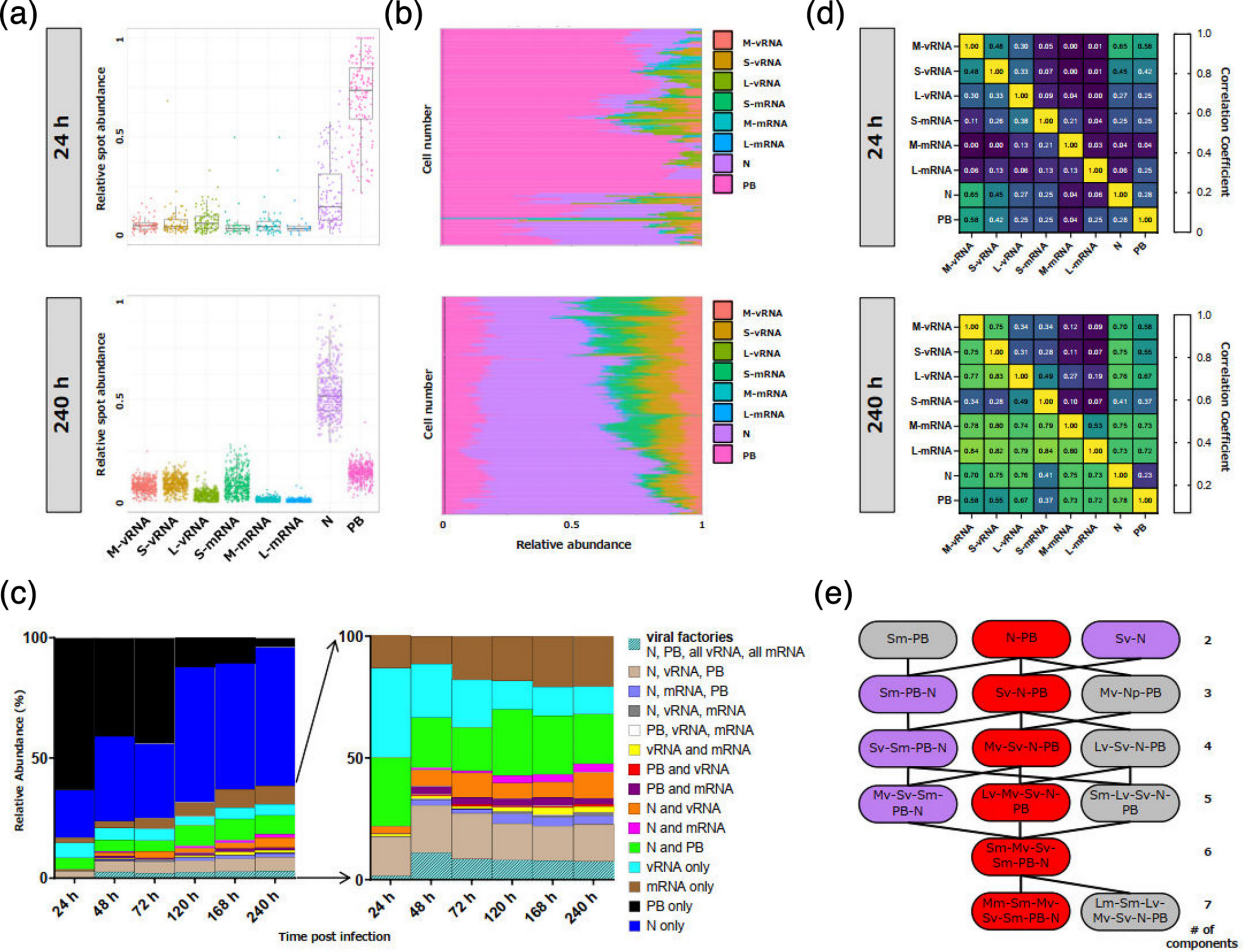
Quantitative analysis of spot ratios, measured by MuSeq-FISH. (**a**) Cells infected with PUUV (MOI=0.3) as shown in Fig. 4 were investigated by MuSeq-FISH and analysed for spot ratios and co-localization of different markers. Mean per-cell spot ratios at 24 and 240 hpi are shown as Tukey boxplots (median, interquartile range and whiskers=1.5× IQR; outliers shown as individual points). Individual cell values are indicated by coloured dots. (**b**) Relative spot ratio analysis at 24 and 240 hpi. Each horizontal line reflects an individual cell, with colours indicating the respective spot species. The number of spots that were detected was counted and normalized to the total number of spots per cell. (**c**) Mean frequency of spots with indicated co-localization relative to all detected spots (relative abundance) at different time points post-infection. In the right panel, N proteins and P-bodies are disregarded to highlight low abundance spot populations. (**d**) Pairwise correlation of fluorescence signals at 24 h and 240 h. (**e**) Potential assembly and clustering pathways in PUUV virus-infected cells. The most abundant multi-colour complexes, with either 2, 3, 4, 5, 6 or 7 components, are displayed in boxes, with lines indicating likely transitions to assemblies with higher complexity. Joining lines were drawn if boxes of adjacent rank (complexity) could be connected to the next through the addition of a single component. Sm, S-mRNA; Mm, M-mRNA; Lm, L-mRNA; Sv, S-vRNA; Mv, M-vRNA; Lv, L-vRNA; PB, P-body; N, N protein.

Next, we analysed all the detected spots together, first focusing on the frequency of individual vRNA and mRNA species, as well as P-bodies and N protein speckles over the course of an infection (Fig. 6a–c). Our data indicate that the large majority (~75 %) of all puncta identified at 24 hpi were P-bodies, whereas N protein aggregates accounted for over 50% after 10 days of infection (Figs 6a–c and S9A). Furthermore, we monitored co-localization of all markers by calculating pairwise correlation coefficients (Figs 6d and S9B). We found a robust correlation between S- and M-vRNA, with N protein and P-bodies at 24 hpi, whereas L-vRNA showed a weaker but still significant correlation. After 10 days of infection, almost all correlation levels increased markedly, except for vRNAs with viral mRNAs, where levels remained comparatively low. We also examined the frequency of different factors alone, or in complexes with each other (Fig. 6c). For this purpose, we did not distinguish between different vRNA and viral mRNA species, meaning that spots containing either of the viral RNA segments were scored as vRNA positive. We observed that the frequency of individual P-body and individual vRNA spots decreased over time (black and cyan), while spots showing co-localization of N protein with either vRNA (orange) or mRNA (pink) co-localization, as well as spots containing only N protein (blue) gradually increased (Fig. 6c). Aggregates containing all components, which could indicate active viral factories, were found to be rare at 24 hpi, peaked at 48 hpi and remained stable but at a low level for the remainder of the time course (Fig. 6c, striped pattern at the bottom). Notably, the frequency of spots containing N protein, vRNA and mRNA, but lacking P-body markers was very low (Fig. 6c, grey bars), supporting the notion that viral mRNA exclusively associates with other viral components in (P-body containing) viral factories and does not directly interact with vRNAs or N protein. Note that a simulated dataset demonstrated the specificity and accuracy for our analysis in identifying actual co-localization rather than random spot clustering as a result of high label densities (Fig. S10).

Ultimately, our aim was to shed light on the orthohantavirus assembly progression and hierarchy. To this end, we assessed the most frequently observed multi-marker spot with a certain level of complexity (single, double, triple, etc. positive) at each time point and utilized this information to build an assembly tree (Fig. 6e). For example, the most probable combination among the double positive spots was N and P-bodies. For triple positive spots, the most abundant combination was N, P and S-vRNA, and the same was true for high order complexes. This approach allowed us to propose an assembly order starting with the association of the N protein and P-bodies, which then incorporates different vRNA species and eventually also encompasses viral mRNAs (Fig. 6e).

### PUUV mRNAs undergo preferential 5′ end degradation in P-bodies

**Fig. 7.**
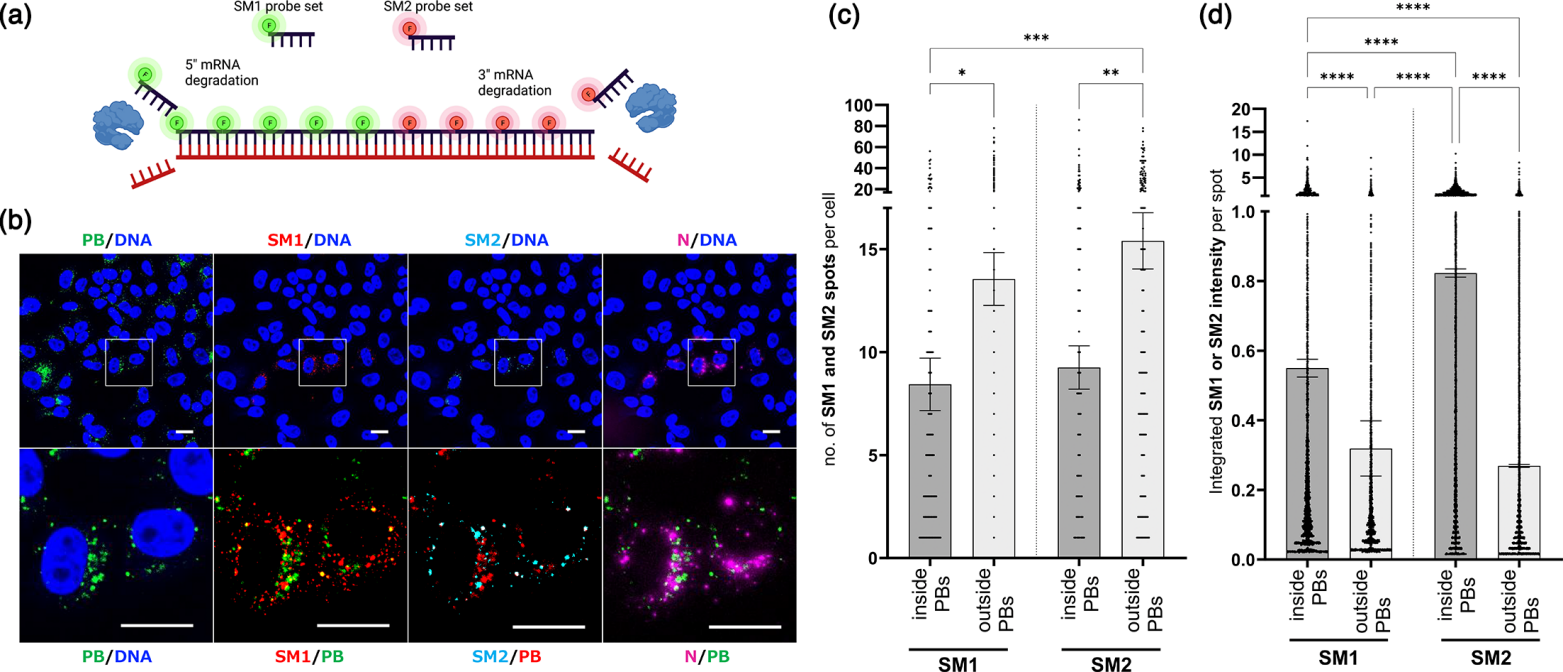
Site-specific labelling of viral mRNA to study their degradation in P-bodies. (**a**) Schematic representation of the experimental setup for mRNA degradation analysis. (**b**) Vero E6 cells were infected with PUUV (MOI=1) and stained at 72 hpi using anti-DCP1a (P-bodies) antibody, accompanied by FISH staining with S-mRNA probe subsets, SM1 and SM2, corresponding to the 5′ and 3′ ends of the S-mRNA. Representative fluorescence microscopy images, including counterstaining for DNA, PUUV N protein and P-bodies using DCP1a antibodies. Scale bars=10 µm. (**c**) Quantification of SM1 and SM2 speckle abundance and integrated signal intensity using CellProfiler. Integrated intensity refers to the sum of pixel intensities within each automatically identified mRNA spot. ****P≤0.0001, ***P≤0.001, **P=0.001 to 0.01 and *P=0.01 to 0.05. All bars indicate the mean with sem. Significance was analysed using one-way ANOVA and Tukey’s multiple comparison tests (*n*>80). PB, P-body.

P-bodies have key functions in the degradation and catabolism of cellular transcripts [16]. There are different mechanisms that digest mRNA from either the 3′ or 5′ ends, ultimately leading to the decay of P-body-associated mRNAs [17]. To test for end-specific degradation of S-mRNA in host cell P-bodies, we devised an approach in which 5′ and 3′ ends of the viral mRNA were labelled with different FISH probes (Fig. 7a), called SM1 and SM2, respectively. If S-mRNA were to be preferentially degraded from either end, we would expect to see differences in the SM1 over SM2 abundance in P-bodies.

We detected both probes in infected samples (Fig. 7b) and quantified the number of SM1 and SM2 speckles, as well as their integrated intensity for either P-body-associated or P-body-independent speckles (Fig. 7c). Firstly, we found that significantly fewer S-mRNA speckles were associated with P-bodies than without for both probe sets (Fig. 7c). In addition, we observed that the overall number of SM1 speckles and SM2 speckles either in or outside the P-bodies was identical for both probes, initially arguing against specific degradation in either direction. Interestingly, the integrated signal intensity of both probes was significantly higher in P-body positive than in P-body negative speckles, reflecting the local enrichment of viral mRNAs in P-bodies (Fig. 7d). Notably, SM1 seemed to have a significantly lower signal intensity in P-bodies than SM2, suggesting preferential degradation from the 5′ end of the transcript (Fig. 7d). This argument is supported by the fact that outside of P-bodies, both SM1 and SM2 exhibited comparable fluorescence intensities. Therefore, it is unlikely that differences in the fluorescence properties of SM1 and SM2 labels account for the lower signals of the P-body associated SM1 probes.

## Discussion

In this study, we quantitatively assessed the spatiotemporal dynamics of infection by the orthohantavirus PUUV using multicolour FISH and IF staining. We monitored all three genomic vRNA segments and viral mRNAs throughout complete infection cycles and concomitantly analysed their co-localization with the viral N protein, host actin, microtubules and P-bodies (Fig. 3).

### Co-localization of vRNAs with host factors and cellular remodelling upon infection

Previous studies have extensively investigated the role of cytoskeletal components in orthohantavirus infection [6,7, 12, 18,20], demonstrating that N-proteins co-localize with microtubules, actin and the intermediate filament protein vimentin [6,12, 18, 20]. Moreover, it is known that a pharmacological disruption of the cytoskeleton can result in the redistribution of viral proteins or the inhibition of key steps of the virus replication cycle [7,12, 18, 19].

Our current experiments firstly indicate a spatial correlation between filamentous actin and vRNAs and the N protein. This suggests that there are direct interactions between viral components and host cytoskeletal factors (Fig. 3). We also observed that both microtubules and actin filaments undergo a marked redistribution upon PUUV infection, with these cytoskeletal elements becoming enriched in the perinuclear region of infected cells (Figs 3a and S5). Moreover, we found that P-bodies exhibit extensive co-localization with N protein and vRNAs, reflecting their crucial involvement in orthohantavirus replication (Fig. 3d). Notably, the S-segment vRNA exhibited a higher frequency of P-body co-localization compared to M- and L-segments. Given that the S-segment encodes the highly abundant N protein, which itself strongly associates with P-bodies, this may reflect preferential trafficking or stabilization of the S-segment in these compartments. While technical differences in probe performance cannot be fully excluded, the segment-specific co-localization likely reflects biologically meaningful differences.

**Fig. 8.**
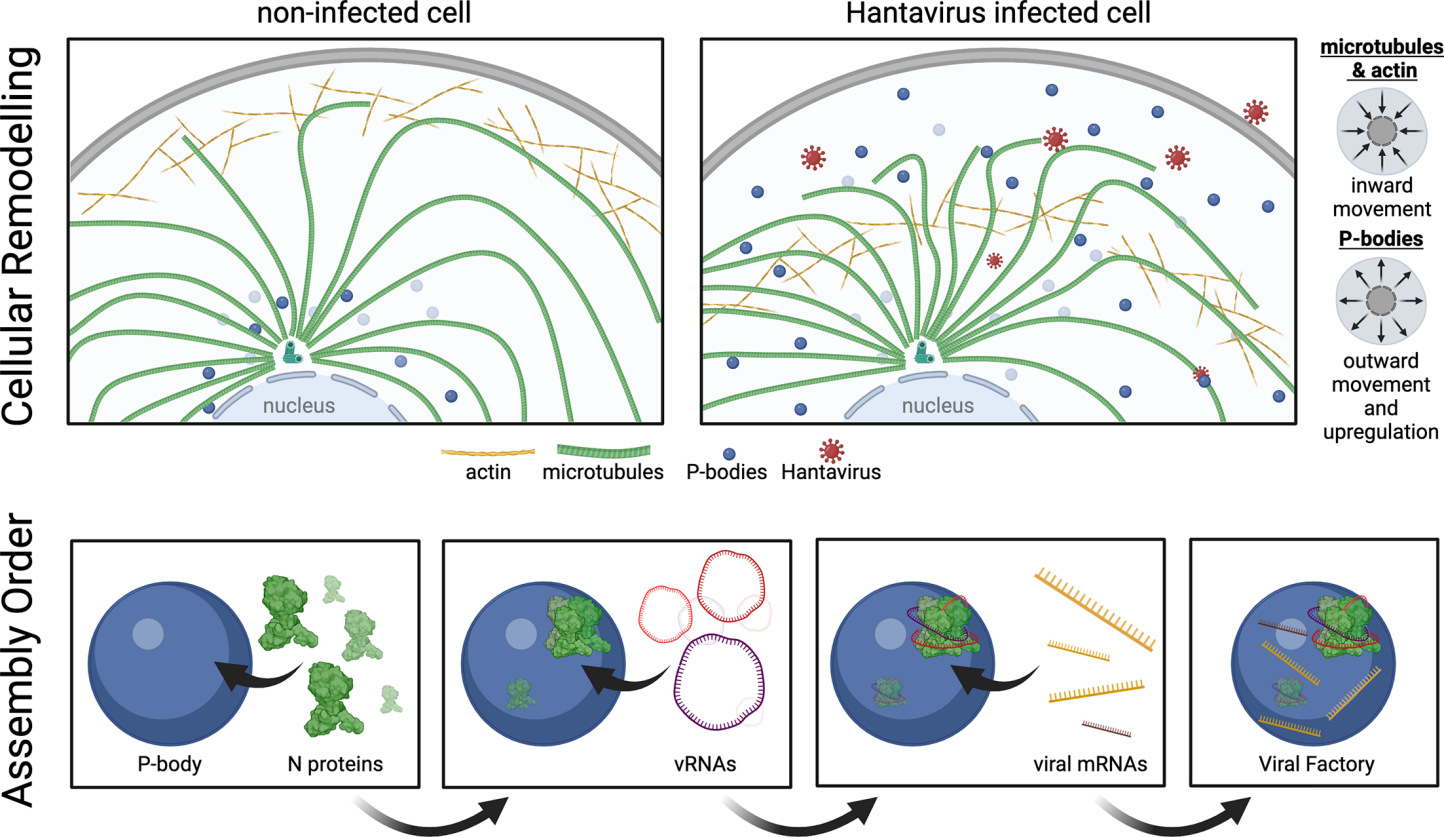
Graphical summary of cellular remodelling upon orthohantavirus infection and a putative orthohantavirus replication complex assembly regimen. Based on our experimental results, we propose a model in which PUUV infection induces a relocation of cellular actin and microtubules towards the cell centre, whereas P-bodies exhibit an outward movement and increase in overall abundance. Moreover, our data suggest that virus factories can form in infected cells through a stepwise association of N protein with P-bodies, followed by recruitment of vRNAs and eventually incorporation of viral transcripts.

In addition to that, PUUV infection was found to induce two major changes in P-body behaviour: (i) an increase in the number of P-bodies per cell and (ii) a redistribution from the perinuclear area towards more peripheral cytoplasmic regions (Figs 1c and 3a, b). We refer to these changes collectively as P-body remodelling, reflecting a virus-induced reorganization of this RNA regulatory compartment (graphically summarized in Fig. 8). Strikingly, significant changes in actin and P-body localization and abundance were observed not only in Vero E6 cells, but also in primary, human endothelial cells (Fig. S5), suggesting that cellular remodelling is a fundamental PUUV-dependent process across different host species and cell types.

Cellular remodelling has also been documented for infection by other orthohantaviruses: Andes virus (ANDV) induces vimentin rearrangement [12], and chronic/late Tula virus (TULV) infection recruits stress granules to filamentous N protein aggregates [6]. Furthermore, we have recently shown that PUUV can induce a redistribution of filamentous actin in infected cells [7], an observation that has been confirmed and expanded upon in this study. We would like to point out that a remodelling of P-bodies has not previously been reported; however, a transient increase in the abundance of stress granules has been observed for both PUUV and ANDV [21], which is reminiscent of the changes in P-body homeostasis that we observed.

Although the consequences of such cellular remodelling remain unclear, we have previously reported that orthohantaviruses exploit and induce macropinocytic fluid-phase uptake even prior to virus entry [7], raising the possibility that actin rearrangement contributes to the manipulation of cellular functions. Because N protein has been reported to co-localize strongly with both actin and P-bodies, even in the absence of other viral components [20], direct interactions between N and these cellular factors may contribute to their redistribution.

Nonetheless, it remains elusive how exactly cellular protein and mRNA homeostasis are affected by such virus-induced host cell rearrangements. Pinkham *et al*. have previously provided a comprehensive analysis of the transcriptional changes upon infection with Rift Valley fever virus, a related member of the Bunyaviridae family [22]. The authors report a significant upregulation of several cellular transcripts, which could result from P-body and, consequently, RNA degradation dysfunction. Whether P-body remodelling and (putative) dysfunction are mechanistically connected however, remains an open question.

We have also investigated P-body-associated RNA degradation processes using a novel approach involving 3′ and 5′ vRNA probe sets. Our results suggest that S-mRNA degradation occurs preferentially from the 5′ end (Fig. 7); however, further detailed studies are necessary to conclusively address the degradation of virus mRNAs. Of note, a number of early studies have thoroughly investigated the role of P-bodies in orthohantavirus infection [4,5]. Noteworthily, the crucial viral cap-snatching, taking place in these cellular entities, is not only a hallmark of the orthohantavirus genus, but a mechanism that is shared across the entire Bunyavirales order.

Importantly, while we observed extensive co-localization of viral RNAs and proteins with P-bodies, our data do not allow us to conclude active genome replication within these structures. Instead, they likely reflect regulatory functions such as cap-snatching, RNA sorting or degradation. This is supported by our 5′/3′ end-specific FISH analysis which indicates that mRNA degradation could occur in P-bodies. Previous studies have shown that the hantavirus N protein binds to and protects host mRNA caps within P-bodies, providing primers for polymerase activity. However, they have not confirmed active replication in these compartments [4,13]. Additionally, work on TULV [6] and Bunyamwera virus [11] supports the Golgi apparatus as the likely site of viral replication, which is now considered a plausible scenario for different orthohantaviruses.

### Spatiotemporal analysis of orthohantavirus RNA complex formation

To characterize the interplay between viral RNA species and proteins, we analysed intracellular complexes containing vRNA, mRNA and the N protein. Throughout the infection process, we observed discrete intracellular foci containing different combinations of these markers (Figs 1, 3 and 6), indicating the coexistence of multiple assembly and replication stages within infected cells. Our analyses suggest disparities in the intracellular distribution between different virus mRNAs on one hand and between virus mRNAs and corresponding vRNAs on the other hand (Fig. 5). Of note, early infection time points exhibited a peak in vRNA, but not mRNA distance from the cell centre, likely reflecting the virus inoculum (Fig. 5). In general, spot ratio analysis further showed a temporal shift in the frequency of different spot species (Fig. 6). Our co-localization analysis also revealed a strong correlation between S- and M-vRNA with N protein and P-bodies already at early time points, with co-localization of all markers increasing at later stages of infection (Fig. 6). Overall, we found that N protein-positive speckles dominated at late infection time points (Fig. 6). Our quantification also revealed that, over time, P-bodies become heavily associated with viral components, with as few as 10% not being associated with either vRNAs or the N protein after 10 days of infection (Fig. S9).

Interestingly, our immunofluorescence analyses consistently revealed the N protein in punctate structures rather than filamentous aggregates, even at 72 hpi. Although filamentous patterns have been reported in other hantavirus models, such as in chronic infection or stress granule-associated contexts (e.g. Davies *et al*. [6]), these morphologies are not universally observed. The absence of filamentous N protein in our study likely reflects the acute infection stage we investigated, the use of Vero E6 cells and the specific experimental conditions employed.

Based on the most abundant multi-marker combinations from our MuSeq datasets, we propose a stepwise assembly pathway (Figs6e  8): viral complexes initially form via N protein association with P-bodies, followed by recruitment of vRNA species and eventually virus mRNAs (Figs6e  8). As virus mRNAs are not predominantly incorporated into virus particles, aggregates that are positive for all markers likely correspond to active viral factories, as previously described for Bunyamwera viruses [11]. Importantly, our assembly pathway indicates a successive recruitment of the different vRNA species, progressing from the S-segment to the M- and then to the L-vRNA. This could suggest that vRNAs are added in the order of increasing complexity (Figs6e  8).

It is important to point out that our study did not attempt to provide a quantitative, molecular analysis of virus particles or intracellular assembly sites. Instead of assessing the composition of such entities at a single molecule level, we aimed to visualize and quantify the interplay between different virus components and their association with cellular factors such as P-bodies or cytoskeletal proteins. Previous studies have thoroughly investigated and described the packaging and intraviral organization of different Bunyaviruses [23,26], which will likely also translate into the orthohantavirus genus to some extent. Nonetheless, further studies are needed to reveal whether packaging dynamics and kinetics are shared across the Bunyavirus order or vary between species.

Collectively, our observations suggest that orthohantavirus infection reshapes cellular RNA processing and structural networks in a coordinated manner. The selective enrichment of S-segment RNA in P-bodies, together with evidence for 5′-directed RNA decay, points to a regulated interaction between viral transcripts and host mRNA-turnover machinery. The parallel remodelling of actin and microtubules across distinct cell types suggests a conserved reorganization of the intracellular scaffold that may define sites of replication and assembly. By linking the localization, degradation and cytoskeletal rearrangement of RNA at single-cell resolution, this work provides a basis for mechanistic studies of how orthohantaviruses integrate these processes to ensure efficient replication.

### Limitations of this study

A key methodological limitation of our study relates to the spatial resolution of fluorescence microscopy. While we employed confocal microscopy with z-stack imaging to investigate the distribution and co-localization of viral and host cell components, the axial (z) resolution remains substantially lower than the lateral (x–y) resolution. As such, neither z-stack-based analyses nor maximum intensity projections can definitively resolve true molecular co-localization, particularly in densely labelled regions. Nevertheless, our use of multiple analytical strategies – line scans, pixel-wise correlation and 3D spot quantification – yields robust and reproducible insights into the spatial organization of viral replication components. These limitations are intrinsic to diffraction-limited imaging and do not compromise the broader conclusions regarding the dynamic association and redistribution of viral and cellular factors during orthohantavirus infection. It is also worth mentioning that, while the multiplex FISH procedure used in this study is theoretically capable of detecting individual RNA molecules, it was not explicitly optimized or calibrated for absolute single-molecule sensitivity. The resulting quantifications therefore represent relative measures of detectable viral RNA abundance rather than total RNA copy numbers per cell.

Another limitation relates to the potential cross-hybridization of FISH probes targeting viral mRNAs. Although these probes are designed to detect positive-sense transcripts, they could also hybridize to antigenomic cRNAs, which share the same polarity [3]. While our FISH approach cannot distinguish between mRNA and cRNA, the observed accumulation of positive-sense signals most likely reflects ongoing RNA amplification, including cRNA synthesis, rather than primary transcription from incoming genomes. Therefore, we interpret these data as evidence of active replication rather than *de novo* input RNA detection [3]. Our vRNA probes, by contrast, are specific to negative-sense genomic RNA and are not expected to bind cRNA or mRNA.

Beyond inherent limitations of fluorescence microscopy and probe design, our analyses focused primarily on Vero E6 cells and exclusively investigated the cell-culture-adapted PUUV Sotkamo strain. This strain may differ from human or rodent infections. Accordingly, the observed remodelling events should be regarded as model-based phenomena that warrant confirmation in additional host-cell systems. Moreover, our FISH-based approach provides correlational rather than functional evidence for P-body involvement. For instance, we cannot conclusively determine whether the observed increase in P-body abundance is driven by specific viral mechanisms or represents a generalized cellular stress response to infection. The enrichment of P-bodies specifically in infected (but not bystander) cells, and their spatial association with viral RNAs and proteins, suggests a degree of virus specificity. However, further studies will be required to disentangle direct viral effects from broader stress-induced remodelling of RNA regulatory compartments.

## Supplementary material

10.1099/jgv.0.002220 Uncited Supplementary Material 1.

## Abbreviations

ANDV: Andes virus
FISH: fluorescence in situ hybridization
hpi: h post-infection
MFrI: mean fractional intensity
MuSeq-FISH: Multiple Sequential FISH
PUUV: Puumala virus
TULV: Tula virus
vRNA: viral genomic RNA
