# Supplementary material for: Remodelling of P-bodies and the cytoskeleton by Orthohantavirus puumalaense (Puumala virus)
Source: J Gen Virol. 2026 Feb 12;107(2):002220. doi: 10.1099/jgv.0.002220 (PMC12900389; doi:10.1099/jgv.0.002220)
Supplement: Uncited Supplementary Material 1. [file jgv-107-02220-s001.pdf]

SUPPLEMENTARY DATA:

REMODELING OF P-BODIES AND THE CYTOSKELETON BY ORTHOHANTAVIRUS PUUMALAENSE (PUUMALA VIRUS)

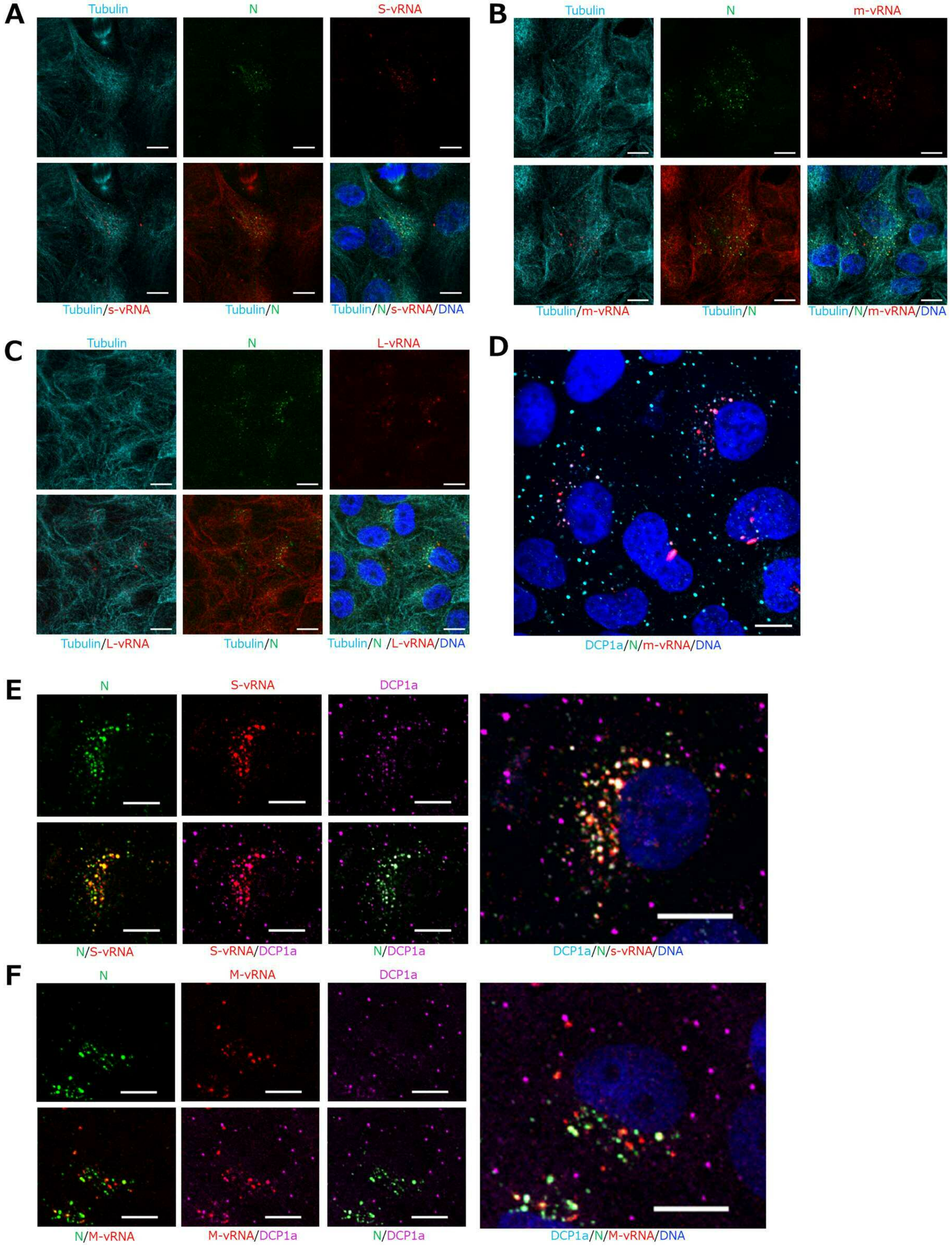

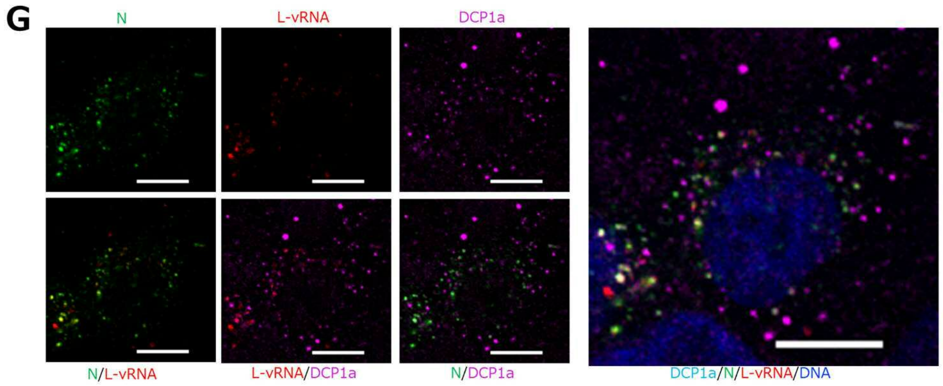

Figure S1: vRNA co-colocalization with cellular markers (related to Figure 2**)**. Additional examples for stainings shown in Figure 2. **(A)** Immunofluorescence staining with anti-tubulin (microtubules) and anti N protein antibodies in combination with FISH labeling of S-vRNA, **(B)** M-vRNA, or **(C)** L-vRNA. **(D)** Anti-DCP1a (P bodies), N protein and FISH staining for S-vRNA in VeroE6 cells infected with PUUV for 72 h. **(E, F, G)** Additional examples of a DCP1a, N protein and FISH-staining for S-vRNA, M-vRNA and L-vRNA, respectively. All images show equatorial slices obtained by confocal microscopy. Hoechst 33342 was used as a DNA counter staining (blue). Scale bars=10 µm.

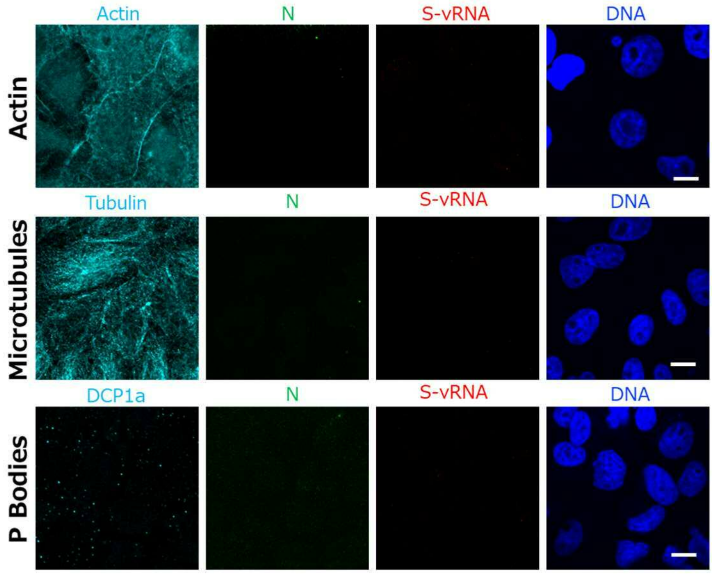

Figure S2: Parallel vRNA FISH and immunofluorescence staining for cellular factors in uninfected cells (related to Figure 2). Combination of TRITC-phalloidin (actin), anti-α-tubulin (microtubules), anti-DCP1a (P-bodies) and N proteins staining with FISH in uninfected VeroE6 cells. Images show equatorial slices obtained by confocal microscopy. Hoechst 33342 was used as a DNA counter staining (blue). Scale bars=10 µm.

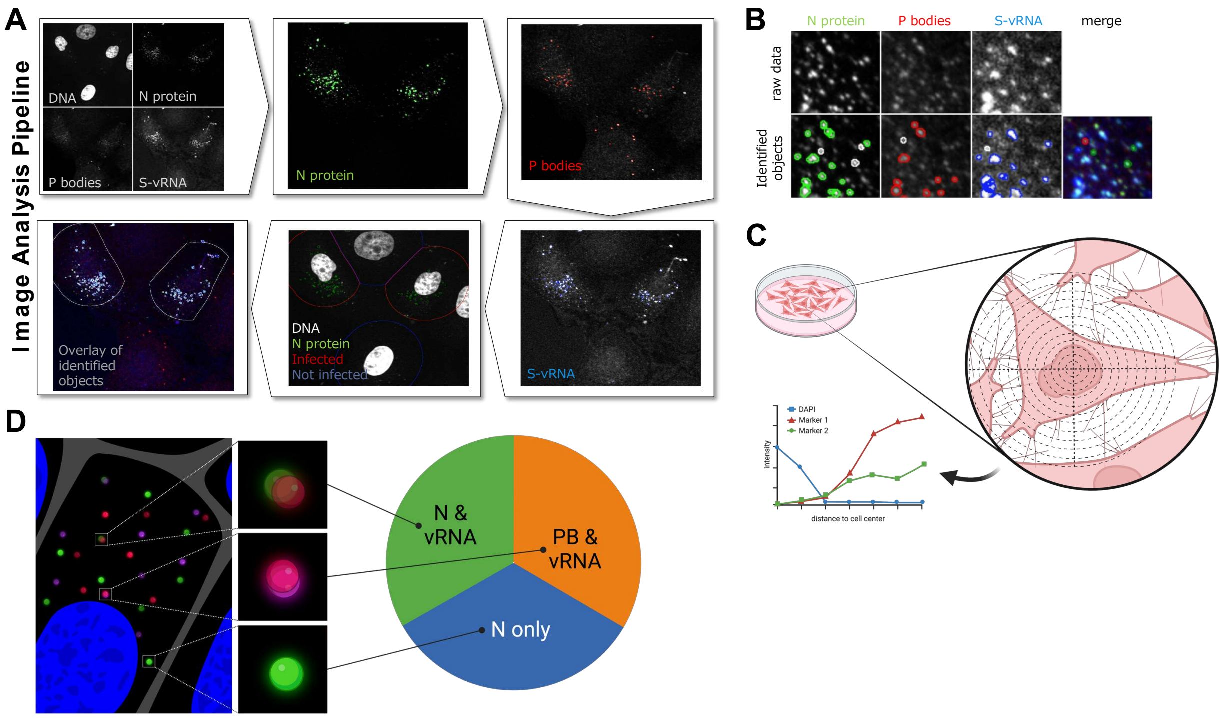

Figure S3: Image segmentation and analysis (related to Figure 3). **(A)** Schematic representation of our custom-made image segmentation pipeline. Briefly, nuclei are identified in DNA counter stainings, followed by recognition of the cytoplasm in IF staining's of abundant cellular markers or in transmission light images. Finally, P-body, N protein or vRNA puncta were identified independently. **(B)** Magnification of representative spot detections conducted by our image segmentation pipeline. **(C)** Schematic representation of a radial distribution analysis, which quantifies fluorescence intensities as a function of their distance from the center of individual cells. Briefly, the analysis first defines radial bins, then sums the overall intensities from equivalent bins of multiple cells and finally displays them in line graphs. **(D)** Schematic representation of co-localization analysis. Each identified spot is analyzed for co-localization of different fluorescence signals and summed up on a per cell basis. Frequencies of different spot species (single positive, double positive, triple positive) are then calculated for all analyzed cells and displayed in pie charts.

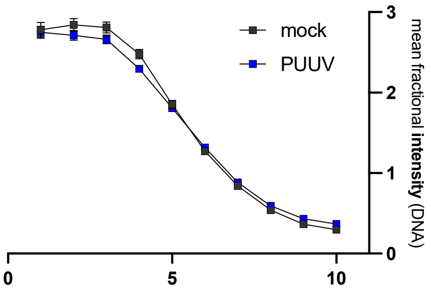

Figure S4: Radial distribution analysis for DNA stainings in infected and non-infected cells (related to Figure 3)**.** Mean fractional intensity was calculated using the CellProfiler module MeasureObjectIntensityDistribution, binning the cell body into 10 section starting from the center of the nucleus going outwards. Several dozen to several hundred cells from different fields of view were analyzed for each plot. Error bars show the standard error of the mean (SEM).

Table S1: Statistics related to Figure 3C. Significance was analyzed using one-way ANOVA and Tukey's multiple comparisons test. ****, p≤ 0.0001.; ***, p≤0.001; **, p= 0.001 to 0.01; *, p= 0.01 to 0.05.

Tukey's multiple comparisons test	Summary	Adjusted P Value
Actin_DNA vs. Actin_N	****	<0.0001
Actin_DNA vs. Actin_vRNA	****	<0.0001
Actin_DNA vs. DNA_N	ns	0.2546
Actin_DNA vs. DNA_vRNA	ns	0.9434
Actin_DNA vs. N_vRNA	****	<0.0001
Actin_DNA vs. DNA_Tubulin	ns	>0.9999
Actin_DNA vs. N_Tubulin	****	<0.0001
Actin_DNA vs. Tubulin_vRNA	****	<0.0001
Actin_DNA vs. DNA_PB	****	<0.0001
Actin_DNA vs. N_PB	****	<0.0001
Actin_DNA vs. PB_vRNA	****	<0.0001
Actin_N vs. Actin_vRNA	ns	>0.9999
Actin_N vs. DNA_N	****	<0.0001
Actin_N vs. DNA_vRNA	****	<0.0001
Actin_N vs. N_vRNA	****	<0.0001
Actin_N vs. DNA_Tubulin	****	<0.0001
Actin_N vs. N_Tubulin	ns	>0.9999
Actin_N vs. Tubulin_vRNA	ns	0.9861
Actin_N vs. DNA_PB	ns	0.9985
Actin_N vs. N_PB	****	<0.0001
Actin_N vs. PB_vRNA	ns	0.2748
Actin_vRNA vs. DNA_N	****	<0.0001
Actin_vRNA vs. DNA_vRNA	****	<0.0001
Actin_vRNA vs. N_vRNA	****	<0.0001
Actin_vRNA vs. DNA_Tubulin	****	<0.0001
Actin_vRNA vs. N_Tubulin	ns	>0.9999
Actin_vRNA vs. Tubulin_vRNA	ns	>0.9999
Actin_vRNA vs. DNA_PB	ns	0.9785
Actin_vRNA vs. N_PB	***	0.0002
Actin_vRNA vs. PB_vRNA	ns	0.5081
DNA_N vs. DNA_vRNA	ns	0.8236
DNA_N vs. N_vRNA	****	<0.0001
DNA_N vs. DNA_Tubulin	*	0.0313

Tukey's multiple comparisons test	Summary	Adjusted P Value
DNA_N vs. N_Tubulin	****	<0.0001
DNA_N vs. Tubulin_vRNA	****	<0.0001
DNA_N vs. DNA_PB	**	0.0014
DNA_N vs. N_PB	****	<0.0001
DNA_N vs. PB_vRNA	****	<0.0001
DNA_vRNA vs. N_vRNA	****	<0.0001
DNA_vRNA vs. DNA_Tubulin	ns	0.7883
DNA_vRNA vs. N_Tubulin	****	<0.0001
DNA_vRNA vs. Tubulin_vRNA	****	<0.0001
DNA_vRNA vs. DNA_PB	****	<0.0001
DNA_vRNA vs. N_PB	****	<0.0001
DNA_vRNA vs. PB_vRNA	****	<0.0001
N_vRNA vs. DNA_Tubulin	****	<0.0001
N_vRNA vs. N_Tubulin	****	<0.0001
N_vRNA vs. Tubulin_vRNA	****	<0.0001
N_vRNA vs. DNA_PB	****	<0.0001
N_vRNA vs. N_PB	ns	0.9248
N_vRNA vs. PB_vRNA	ns	0.9898
DNA_Tubulin vs. N_Tubulin	****	<0.0001
DNA_Tubulin vs. Tubulin_vRNA	****	<0.0001
DNA_Tubulin vs. DNA_PB	****	<0.0001
DNA_Tubulin vs. N_PB	****	<0.0001
DNA_Tubulin vs. PB_vRNA	****	<0.0001
N_Tubulin vs. Tubulin_vRNA	ns	0.9579
N_Tubulin vs. DNA_PB	ns	0.9925
N_Tubulin vs. N_PB	****	<0.0001
N_Tubulin vs. PB_vRNA	ns	0.1894
Tubulin_vRNA vs. DNA_PB	ns	0.7265
Tubulin_vRNA vs. N_PB	***	0.0002
Tubulin_vRNA vs. PB_vRNA	ns	0.7102
DNA_PB vs. N_PB	****	<0.0001
DNA_PB vs. PB_vRNA	ns	0.1016
N_PB vs. PB_vRNA	ns	0.6580

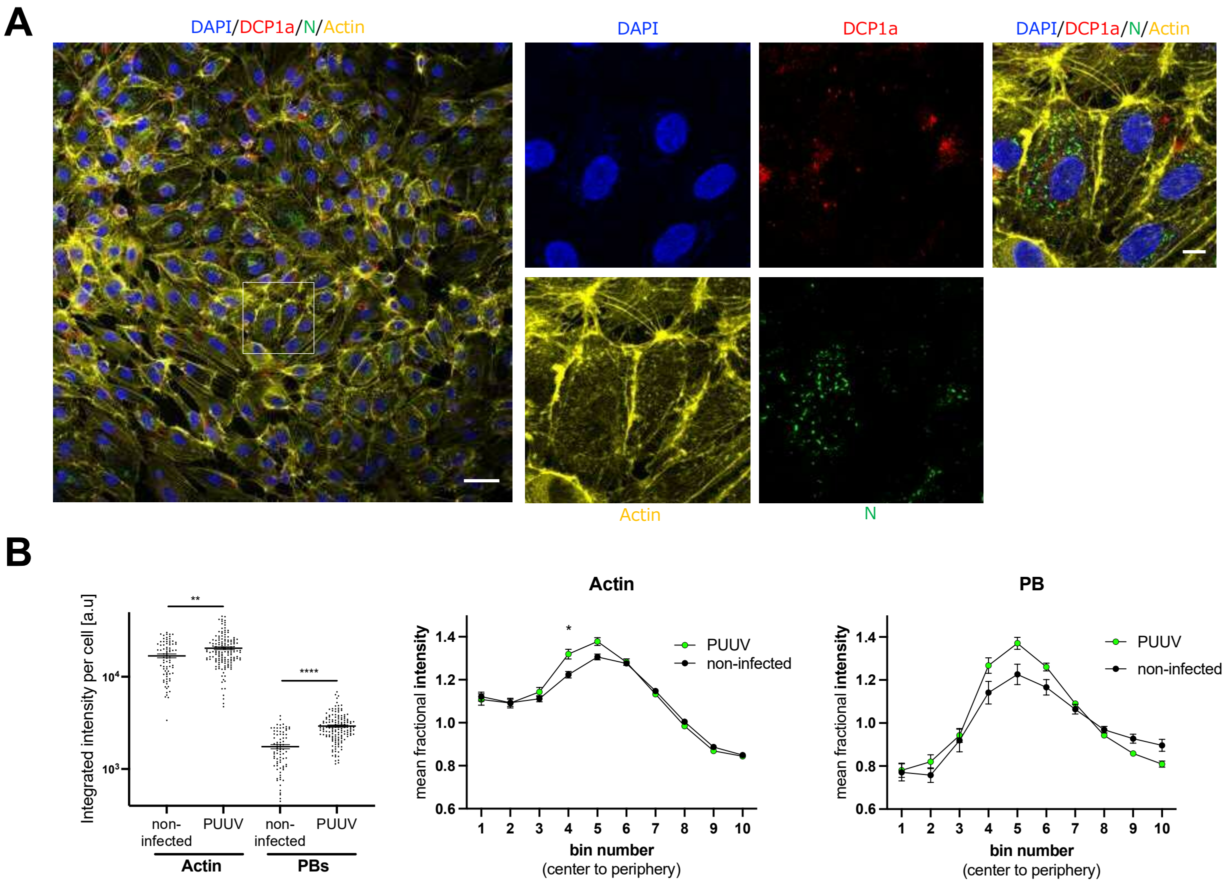

Figure S5: Cellular remodeling of human pulmonary microvascular endothelial cells (related to Figure 3)**.** Cells were infected with PUUV (MOI 0.5) and subjected to immunofluorescence staining at 72 hpi. Then, samples were imaged by confocal microscopy and analyzed using CellProfiler. (A) Representative fluorescence microscopy images. Marker colors are indicated above and below the micrographs. Scale bars=10 µm. (B) Quantitative image analysis using Cell Profiler. Left: integrated Actin and PB intensities were assessed in both infected and non-infected samples on a per-cell basis. Each dot represents individual cells and error bars show SEM. Significance was assessed by unpaired Student's t test. Center and right: Mean fractional intensity (MfrI) was calculated using the Cell Profiler module MeasureObjectIntensityDistribution, binning the cell body into 10 sections starting from the center of the nucleus going outwards. Several dozen to several hundred cells from different fields of view were analyzed for each plot. Error bars show the standard error of the mean (SEM), and statistical significance was assessed by comparing PUUV and non-infected samples at each bin.

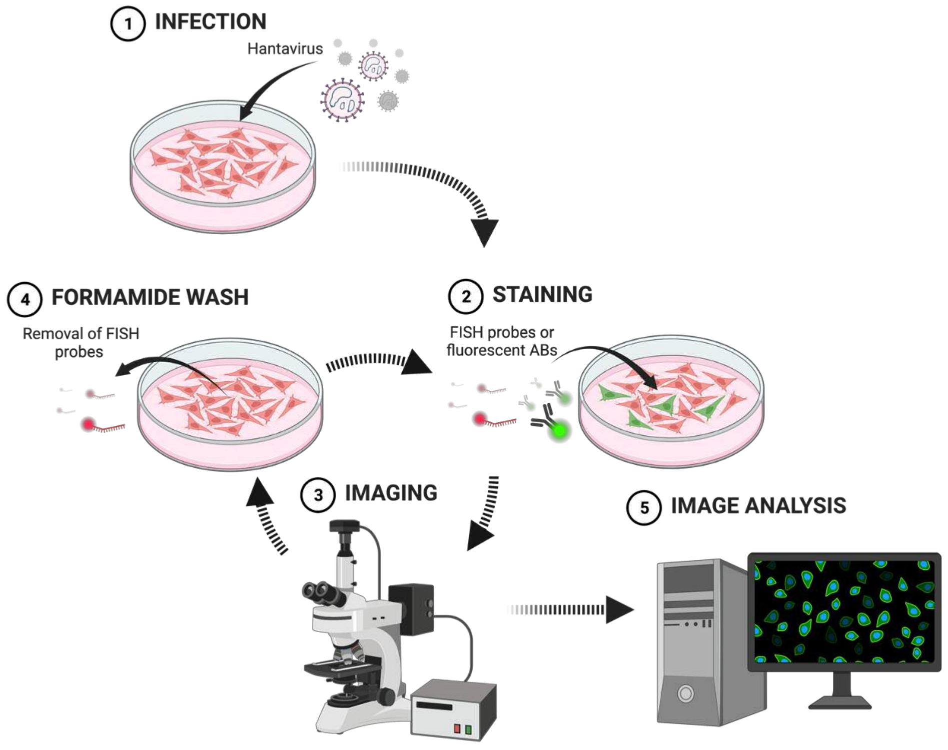

Figure S6: MuSeq-FISH working principle (related to Figure 4)**.** (1) Cells are infected with Orthohantaviruses and subjected to (2) FISH staining, followed by (3) confocal fluorescence microscopy and (4) a Formamide wash that removes all FISH probes. Subsequently, another round of FISH staining can be conducted.

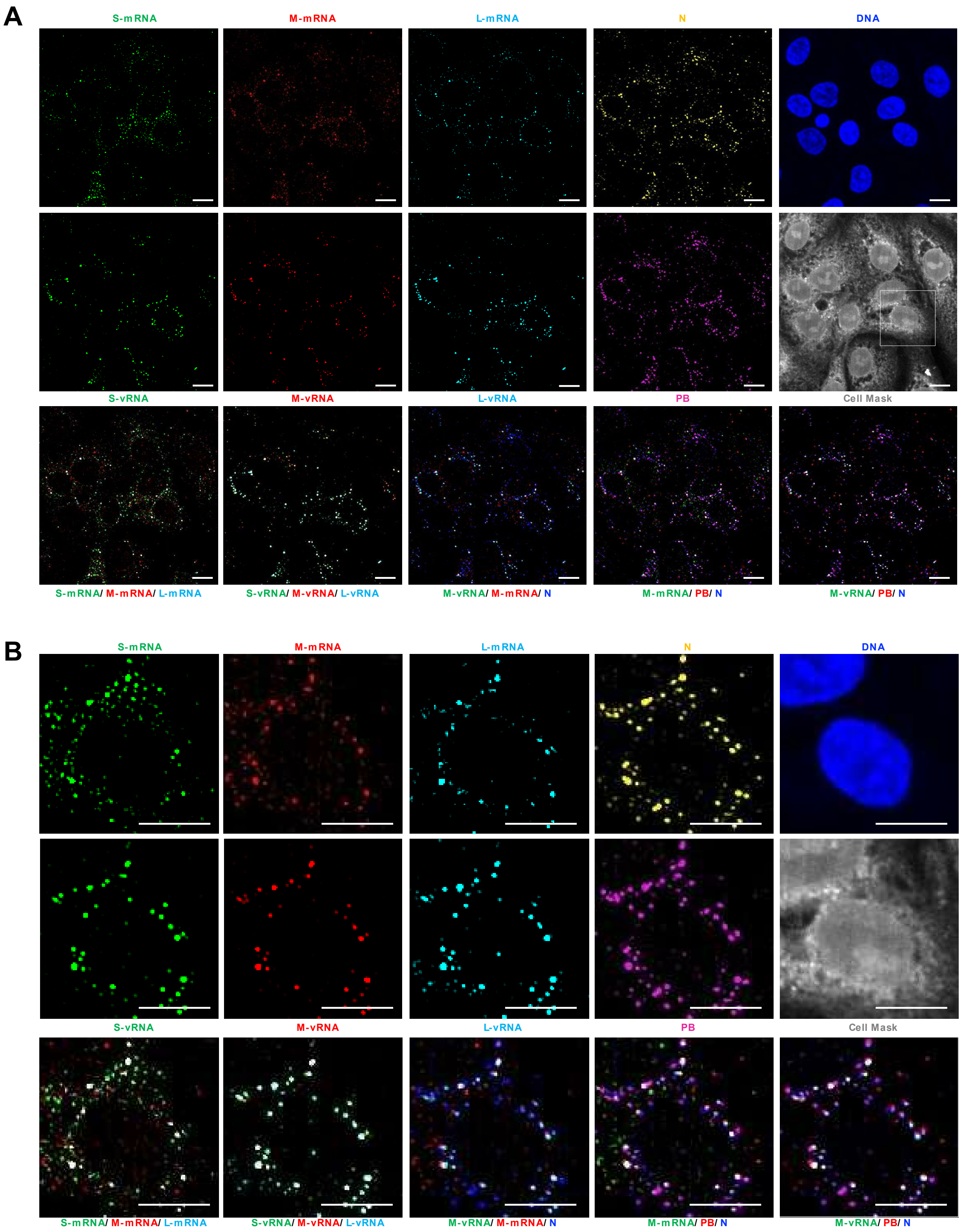

Figure S7: Imaging of PUUV vRNAs and their transcripts in infected cells (related to Figure 4). VeroE6 cells were infected with PUUV for 240 h and analyzed using MuSeq-FISH. (A) Overview and overlay images of a representative region of interest,

showing each staining individually: S-mRNA and S-vRNA in green, M-mRNA and M-vRNA in red, L-mRNA and L-vRNA in cyan, N protein in yellow, P-bodies (PB) in purple, DNA in blue and cell mask staining in grey. Overlays display stained factors as indicated below. (B) Magnification of the boxed region shown in the cell mask image in (A). Scale bars=10 µm. Images represent maximum intensity z projections.

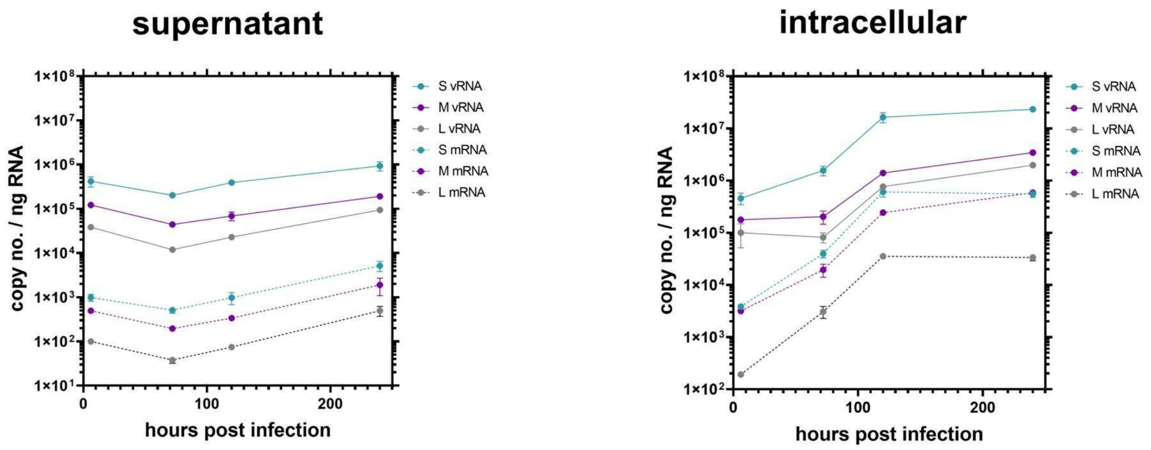

Figure S8: Quantification and kinetic analysis of all PUUV viral mRNA and vRNA species at different time points post infection using qRT-PCR. Vero E6 cells were infected with PUUV MOI 0.3. Cells and supernatant were harvest at the indicated time points. Both graphs show the mean RNA levels with the SEM. The samples were done in triplicates.

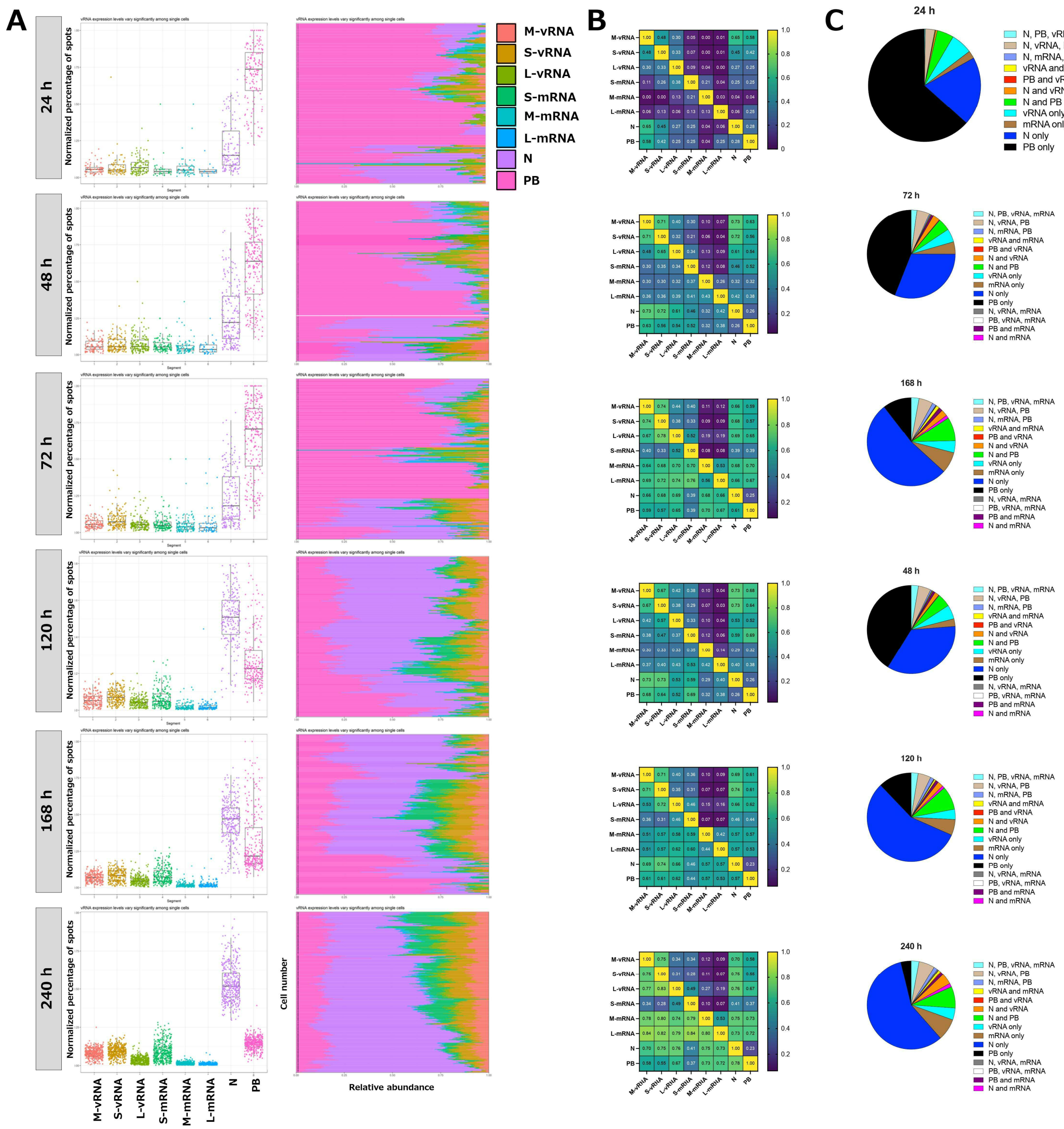

Figure S9: Quantitative analysis of spot ratios as measure by MuSeq-FISH (related to Figure 6)**. (A)** Mean per cell spot ratios and relative spot ratio for all time points post infections are shown. Per cell spot ratios are displayed in a Tukey boxplot representation. Upper bound of box, center and lower bound of the box represent the 75th percentile, the 50th percentile (median) and the 25th percentile, respectively. Upper and lower whiskers represent the maxima and minima of the boxplots showing the respective largest or smallest value within 1.5× interquartile range above the 75th or below the 25th percentile (bounds of box). Individual cell values are indicated by colored dots. Relative spot ratios are shown in horizontal lines, reflecting an individual cell, with colors indicating the respective spot species. The number of spots that were detected was counted and normalized to the total

number of spots per cell. **(B)** Pair-correlation of fluorescence signals at 24h and 240h. **(**C) Relative abundance of different spot species at different time points post infection. Individual pies indicate the fraction of a certain spot species (see legend).

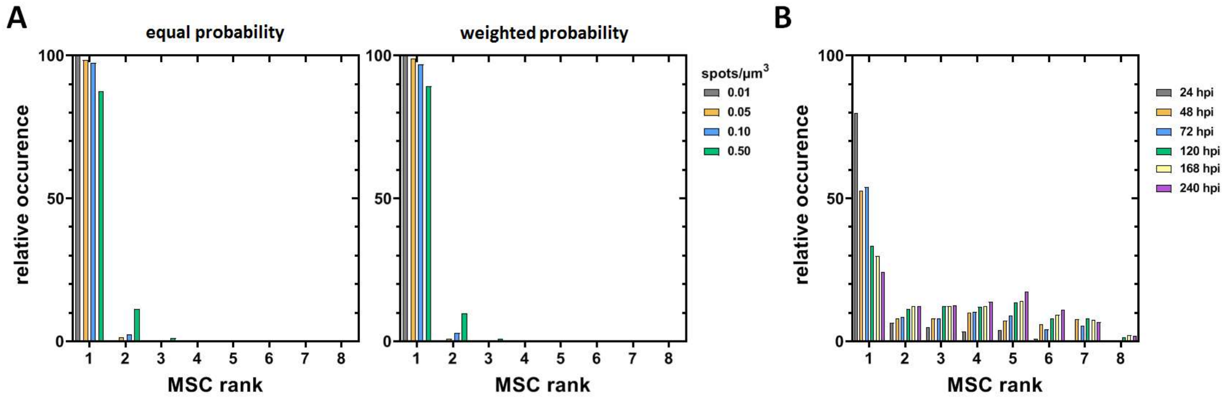

Figure S10: Co-localization analysis of a simulated data set and a MuSeq-FISH data set of PUUV infected Vero E6 cells. To ensure accurate MSC grouping and proper assessment of colocalization data, an artificial data set was created. This data set consisted of a simplified cell shape model with a randomized spot distribution. The spot densities chosen for the simulated cells matched the spot densities previously measured in PUUV infected cells. All segments were equally represented in the simulated cells or had a weighted occurrence. Higher spot densities resulted in higher colocalization ranks. Only at a maximum spot density of 0.5 spots/µm3 (equivalent to approximately 750 spots per cell) and with an equal probability of the individual segments in a cell, were higher MSC ranks up to 3 determined with a likelihood of about 12 % **(A)**. The rate of random colocalization in the weighted occurrence of the individual segments was always below 10%, primarily due to the clustering of PB and N. Contrary to the simulation, PUUV infected cells showed higher co-localization ranks up to 8 (B), despite having fewer spots compared to the maximum in the simulated data set (Figure 7A). Therefore, higher colocalization ranks observed in PUUV infected cells are unlikely to be a result of random co-localization caused by high spot densities.

SUPPLEMENTARY METHODS:

REMODELING OF P-BODIES AND THE CYTOSKELETON BY ORTHOHANTAVIRUS PUUMALAENSE (PUUMALA VIRUS)

PUUV Infection. Briefly, cells were grown to 50-60% confluency, rinsed once with Dulbecco's phosphate-buffered saline with Mg^{2+}/Ca^{2+} (DPBS+/+) and infected with a multiplicity of infection (MOI) of 0.3 - 1 in DMEM containing 1% FBS to reduce fluorescence background in microscopy experiments. After an incubation of one hour at 37°C, cells were washed three times with DPBS+/+ to remove unbound viruses. Cells were further incubated with DMEM supplemented with 1% FBS at 37°C. Fixation, staining and microscopy was performed at 72 hpi if not otherwise stated, to ensure robust hantavirus infection levels and advanced replication stages [8]. All solutions, buffers, and media used for cell culture were purchased from PAN-Biotech (Aidenbach, Germany).

Immunofluorescence and DAPI staining. Cells were incubated with 0.2% acetylated bovine serum albumin (BSA) (B8894, Sigma-Aldrich, St. Louis, MS, USA) in 2× SSC buffer supplemented with 2 mM of the unspecific RNase inhibitor vanadyl ribonucleoside complex (VRC, Sigma-Aldrich, St. Louis, MS, USA) for 15 min at RT, followed by incubation with a cross-reactive anti-Tula virus/Malacky polyclonal N protein antibody raised in rabbits(1:500) [9], anti-Dcp1a for P-body detection (1:100, #ab57654, Abcam, Cambridge, UK), anti-α-tubulin (1:1000, #T5168, Sigma-Aldrich, St. Louis, MS, USA) and secondary IgG antibodies, conjugated with AlexaFluor 488 or 647 (1:1000, Thermo Fisher Scientific, Waltham, MA, USA) at dilutions of 1:100 to 1:1,000 in BSA-containing 2× SSC buffer for 45 min, again at RT. Samples were washed twice to remove unbound antibodies with 2× SSC buffer for 10 min at RT. Subsequently, cells were stained with either 100 nM 4′,6-diamidino-2-phenylindole (DAPI) (Thermo Fisher Scientific, Waltham, MA, USA) or Hoechst 33342 (Thermo Fisher Scientific, Waltham, MA, USA) at RT

for 10 min to label DNA. Finally, the samples were again washed twice with 2× SSC. Filamentous actin was stained with TRITC conjugated phalloidin (#P1951, Sigma-Aldrich, St. Louis, MS, USA) according to the manufacturer's protocol.

FISH Probe Design. The African Green Monkey genomic sequence was received from Dr. Den Kenwar (McGuill University Quebec, Canada). Native PUUV (Sotkamo strain) vRNA sequences were obtained as cDNA transcripts under following accession numbers from GenomeNet [1]: S: NC_005224.1, M: NC_005223.1, L: NC_005225.1. The sequences were used for the design of oligonucleotides complementary to the viral RNA segments and viral transcripts without binding to host cell genome in order to avoid false-positive probe binding. Initially, potential probe sequences targeting PUUV negative stranded viral RNA were pre-selected utilizing StellarisDesigner (Biosearch, Novato, USA). Design of probes was performed with prospect of potential life-cell detection employing forced intercalation probes (FIT-probes). Thus, the selection process based on StellarisDesigner was modified to ensure selection with minimal probe-probe interaction (for FISH probes this aspect is less critical, since interacting probes are eliminated in the following washing procedure). A primary set of possible probe sequences was obtained by splitting a whole viral RNA segment into partially overlapping equal nucleotide (nt) segments and collecting the subsequent StellarisDesigner probe suggestions. By providing the StellarisDesigner with multiple segment packages instead of the entire viral RNA segment, the number of suggested probes increased, allowing for probe selection based on customized criteria. Since the genome of African Green Monkey not available for StellarisDesigner-internal masking, suggestions by StellarisDesigner were based on interactions of probes with human genome at masking level five. This represented the highest stringency the software of StellarisDesigner generally offered to improve probe specificity. Subsequent secondary selection was based on

several demands the sequences, probes as well as targets, had to fulfill. Sequences were filtered by utilizing different software sources with respect to properties of the probe itself, but also to the native state of target sequences. In context of possible secondary structures such as hairpins and self-annealing sequences were filtered via PCRStats [2]. Accessibility to target vRNA sequences was evaluated with the public tool Oligowalk [3], which again is based on the mFold [4], a software for the prediction of nucleotide folding and secondary structure. Free Gibbs energy (ΔG) calculations for intramolecular, homo- and heterointermolecular interactions of all probes were performed by the publicly available web-application NUPACK: The Nucleic Acid Package [5]. Probes predicted to have low ΔG values, were considered more prone to form intramolecular oligo structures or stable oligo-oligo duplexes due to intermolecular interactions. Both would impede hybridization of probe and target, since binding sites would be blocked. Finally, uniqueness of remaining sequences was verified by blasting these against the genome of *Homo Sapiens* (9606), *Canis lupus familiaris* (GCF_000002285.5) and *Chlorocebus aethiops* (African Green Monkey, GCA_023783515.1) via NCBI-Blast [6]. All probe sequences are provided below (Table SM 1, Table SM2 and Table SM3).

Table SM 1: FISH-Probes used in experiments presented in Figure 1-3.

L-vRNA probe sequences, **TAMRA** labelled			
aggtatgtaaactaggtggt	taacctttttagtgtacctgg	cagtatcacctacaaatgct	gcagtttaatatcagaggca
taaagagttgttgcgctgcg	cctaatgactcggtatatgt	cgcaatctttgataatctcc	acgatagatacgttctgggt
gcaggtgttccaaatgatgt	gttacagatagaagctcaga	gtggcctagtagaaggaatg	ggcgttgttcaagcaatgag
tcgtacatttgatattccga	tatagacctcagccattatg	ccatcaacaattgcatctga	ctgagacaccattacagcta
ccaagggtttactttaagcc	aaggagtgttcggtgagttg	gtagcaagtcaagtacagca	aaacaacagcctggcatcta
atccacctagaaacagttga	agtcttaaaggtatgtcagg	tcctacagtacttgaacttt	ctgaggctgatagaggtttt
gcagatgcaactaagtggtc	tttggggcagcaatatcatt	ggagcctatagatgatggta	ggtttcaatatgtgacccaa
gtttaacctccatgaacaca	ggttactttgatgatcttgc	ggttctatgtcaatcatgga	ctgggataggaatggcagat
gtgtttacccctaaaagtga	tatctagagctttggagtga	tgaatgatcccagcatagtt	atgggtgacatttagagagg
agttgctcgtacattcacag	tgatgtattggatagtgcct	aggaggtattaaaggctgta	tatcgaggtttggagatggt
ggttgaaatacttggtgttt	gtgggtagacttattttgtt	tcaagaagtgttgtgttgtt	tttcttcttgcacatgctta

gcagtaggaaatgtgtggtt	gggtttctaaaggtttgtct	tacctactctggctttgaac	cctaatgactcggtatatgt
M-vRNA probe sequences, **Quasar 670** labelled			
tatttgcattatgttgtcca	agcattacttatactatcca	tgggaattggatggttgtgg	aagatctttgatgatggcgc
gtgttatggttctactacaa	ctggtatgtactgttagttt	aacatcagctacagatgggg	atgtgattgtaagccgtgat
acatcagttaaagtgtgctt	ttgcggtgtatatctggata	atgaaactggttggggttgt	gttggactgatactgcacat
ttttgtaggtttagtgtggt	ctatttaggtaccgcagtag	gcagggttgttatcgaacat	ctaacatcacggtttcagga
tggtttgtgaggtatgtcag	catttgcatatctttgctct	ctgggttctgataccaacta	cacattctcttgctgttgag
tcacctacttgcctgataaa	ttctgagacaggtattttca	ccactctatacatctactgg	aaggtattgcattttcaggc
ttctgtcacgaatggctttt	acatctgtcttattgggagt	ctcttgttcagaagactggg	gccaagccatacttatgata
cctagtggttgaaacagcag	taagaggtctgtgcttgatc	gcatcatcaacaagttttca	ggacatgggagacaaaaggt
acttaaaatggagtgtccgc	atgtcagcctactgtttatt	accacatggcgaagatcatg	gtagacatggatatcactgt
agagctgttcaattggttca	atgttgcttgttttagagct	aatttggtgatcccggagat	ggtaaaggtggacattctgg
aaagtgctctagtacagggt	ctgtcccaatacagcaaatt	cctgtatgtcagtttgatgg	gggacctattcaaacatgct
gggaaggttacaggtaaagc	accttagttattggacagtg	cttattgccttaatccatct	ctgctagtgctgagacacaa
S-vRNA probe sequences, **Quasar 670** labelled			
atttatatgtttctatgcct	tccttaattacatatgcact	gcagattggtatacaattgg	aattatcctcttgtttatgt
tctttgcatgtgcaccagac	tcatgagtccagttatgggg	atgcccaccaacatgtatct	atcacttccatcttggtgat
tgcagttatcagggtcatta	tatggatccagagctcagag	attcatttctacctcagtgt	cagggatcatatgtgctagt
actcagatccaggttcgtaa	accctacatcacctgacaac	gccaggatgtgcttgacaaa	aactcacacctggcagattt
gattcgcctaatgcaccatg	agagaaggatggcagatgct	tgcttttcaaggagtctact	gcaacttattgttgccagac
gtatgtgatagggttcacac	tcatgtggcagacattgaca	taagactgttggcacagcag	ttgcacaggcactgatagat
aacgcgtgggagacagactg	gggatcactaatggcagtta	taattgactatgcagcctct	actggaggacaaactcgcag
agtgacttgacagatatcca	agtaaaaccaggcacaccag	ttacctgcgtcgaactcaat	atgggaattcagcttgatca
aggatataacccgccatgaa	cctatcatccttaaagcttt	attgaagaaccaagtggcca	ggacccagatgacgttaata
caagccaggcaacaaacagt	cccagtcaactatgaaagca	taaacctactgacccgactg	tcaatggcataaggagacca
gaatggggcaaagagatggt	gctactacgagaaagtctgg	ggaaaaagagtgcccattca	atatcgaaccaggaaccact
gtagtgagttgttaacatgt	ggcccaaacatgtgaaactg		

Table SM2: FISH-Probes used in experiments presented in Figure 4-6. HybFLAP-Y sequence (TTACACTCGGACCTCGTCGACATGCATT) was added to the 5' end of each probe. nt: nucleotides.

L-vRNA				
**name**	**start**	**end**	**size**	**sequence (3' → 5')**
LvRNA_Y_102	102	131	29	ACTCCCAGACTCCTTCTTTTCATTTAGAC
LvRNA_Y_198	198	230	32	TGTTAAAGGCTGGGGAGAGTGTGTCAAGGATC
LvRNA_Y_457	457	489	32	TACAAGATGAATCTGCTTATACTGGAGATCTG
LvRNA_Y_625	625	657	32	GTTTTACAACAGTGAGCACAATCTCTAGCACA
LvRNA_Y_788	788	820	32	AATTTTATGAAAGGGTTGCCTGCAGCAATTCC
LvRNA_Y_895	895	927	32	TAAAGGATGTGAGATATCAGGCATTACAGGCA

LvRNA_Y_994	994	1026	32	AAGCATTCTCAATTACAATTCAGGCTAATGGC
LvRNA_Y_1031	1031	1059	28	GAACAGGCCTTAGGGTAAGACACCCGAC
LvRNA_Y_1061	1061	1093	32	GTGCCCTGTATAGTTTATAGGAATTGTACTCT
LvRNA_Y_1176	1176	1208	32	AGTAACTAAAGAGTTGTTGCGCTGCGGTATGA
LvRNA_Y_1485	1485	1517	32	ATTTTGTTCAAGAGGGGTATTGCCTATTGACC
LvRNA_Y_1764	1764	1796	32	AGTGCAAGGAAATGTTGAGGGTACAGCTGAGT
LvRNA_Y_1901	1901	1929	28	ACTCCTTATCCACTGATTTGCAACTCAT
LvRNA_Y_2000	2000	2032	32	CGGGATCAAACAATTCAGAATCCTATTACAGC
LvRNA_Y_2189	2189	2217	28	CAGATGCATTTGCGTCTGAATATCGGCC
LvRNA_Y_2219	2219	2251	32	GAATGGGTGACATTTAGAGAGGTTCTTGCTGC
LvRNA_Y_2308	2308	2339	31	AGTTACTGCAATGACAATGCAATCACCATTG
LvRNA_Y_2674	2674	2705	31	TATGTCAATCATGGAGTTAGCAACAGCTGGG
LvRNA_Y_2707	2707	2739	32	ACTTAATACCTATTCCTTTAGGTGGTGATGGT
LvRNA_Y_2824	2824	2854	30	TGTCCACAGCTTGCACAGTTAGGAATAGTG
LvRNA_Y_2889	2889	2919	30	TAGGTTACTTTGATGATCTTGCAGCTGCTC
LvRNA_Y_2921	2921	2953	32	ATCCTTTTAGGTTCTCTATCAGATTTACCTGG
LvRNA_Y_2968	2968	2999	31	GTCTACATTTTTTGAAGGCTGTGCAGTCTCT
LvRNA_Y_3100	3100	3131	31	CTTTCATTGGCATGCTGTGAATCAGGAGATG
LvRNA_Y_3176	3176	3208	32	ATTTATGGTTATCTGGAGCCTATAGATGATGG
LvRNA_Y_3215	3215	3247	32	TTTTTTGAATTTGCCCATCATTCTGATGATGC
LvRNA_Y_3295	3295	3327	32	AGTGTTCTTCATTATTTGGGGCAGCAATATCA
LvRNA_Y_3582	3582	3614	32	GTATGTTAGTGCAGATGCAACTAAGTGGTCAC
LvRNA_Y_3655	3655	3687	32	TTCGATGGGCCTCAGGTGAAAGTGAAATACAG
LvRNA_Y_3755	3755	3787	32	GATTATTATGATGCTATTGCCAAAGTTGTCCC
LvRNA_Y_3898	3898	3930	32	ATCCTACAGTACTTGAACTTTATGAGCAGACA
LvRNA_Y_3991	3991	4023	32	AAGGTATGTCAGGTGCATTACAGGAAGATGGT
LvRNA_Y_4060	4060	4092	32	TGGCAAGCAACATTATGAACAAACACTGGGAT
LvRNA_Y_4128	4128	4160	32	TTGTCAAGAAGTTGTCGAACTAGCTGCTCAAG
LvRNA_Y_4302	4302	4334	32	TGAGAAGGGATTGCATGGCAATCTTACAGAAG
LvRNA_Y_4394	4394	4422	28	GCGGAGTGTATCCCTCTCTTATGTCAAG
LvRNA_Y_4583	4583	4613	30	ATCTACCTACTCTGGCTTTGAACCTTTAAT
LvRNA_Y_4615	4615	4647	32	TTGATAATCTCCGATATTTGATTCCAGCTGTC
LvRNA_Y_4683	4683	4712	29	TCGGTCCGTATTTGCATTCCATTTCCTTC
LvRNA_Y_4714	4714	4746	32	AAGATCAAGGACATTTTCCTCTTCAACATGCA
LvRNA_Y_4973	4973	5005	32	GCATGTGGTAGTGTCTTATTATGCATATTACC
LvRNA_Y_5192	5192	5224	32	CTAGGTGATGTGGCAAATGGATTAGATCAACC
LvRNA_Y_5404	5404	5436	32	AGCCAACACAGAATATTAAAGAACCAGGTACC
LvRNA_Y_5570	5570	5602	32	TCAACAATTGCATCTGATCAGTATGATACACG
LvRNA_Y_5638	5638	5670	32	ATGGACACTCTAGAAAGCCTAGGAATTTTCTT
LvRNA_Y_5801	5801	5829	28	ATTATAGACCTCAGCCATTATGCCAACC
LvRNA_Y_5831	5831	5863	32	ACAGGTCCTAAGGTTCGTACATTTGATATTCC

LvRNA_Y_6146	6146	6177	31	TGACAGCTGATGTGGCAAGGGGAGTTAGAGA
M-vRNA				
**name**	**start**	**end**	**size**	**sequence (3' → 5')**
MvRNA_X_145	145	177	32	TATGCTATATTCGATTTACTAGTGCAGTCACC
MvRNA_X_233	233	265	32	ACTATCCATCTTCTTATTTGCATTATGTTGTC
MvRNA_X_269	269	301	32	TTGGATGGTTGTGGCTGTGTTGATAGCATTAC
MvRNA_X_303	303	335	32	TAGGAGAATGGGTTCTTGGAGTCCTTAATGGG
MvRNA_X_371	371	403	32	AGCTGATAGTGATAAGATCTTTGATGATGGCG
MvRNA_X_425	425	452	27	GGTTAGTTGCAGCTGCACCACACTTGG
MvRNA_X_576	576	608	32	TTTTAACTTCAATTAAGGCATGTCATGCTGCT
MvRNA_X_652	652	681	29	ACAGATGGGGCATGGGGTTCAGGAGTTGG
MvRNA_X_732	732	764	32	ACATCAATGTGATTGTAAGCCGTGATTTGTCC
MvRNA_X_768	768	800	32	TGGAGTGGATTGACCTAGATAGTTCACTTCGG
MvRNA_X_802	802	834	32	AATGTAACAGAACCACACATCTCTACAAGTGC
MvRNA_X_971	971	1000	29	TCCCGGAGATATAATGAGTACACCCACAG
MvRNA_X_1002	1002	1034	32	AGCAATGGTGTACTACTACATGTCAATTTGGT
MvRNA_X_1062	1062	1089	27	CCTTCTGATACCCTCTTATTCCTCGGT
MvRNA_X_1091	1091	1123	32	AGTGTGCTTGATAGGTACCATTTCAAAGTTCC
MvRNA_X_1177	1177	1209	32	ACAAGGAAAGTATGCATTCAGTTGGGTACCGG
MvRNA_X_1281	1281	1308	27	GATTGTCCTGGTGTAGGGACAGGCTGC
MvRNA_X_1454	1454	1486	32	ACAGGTGATTCATGCTGAAATTCAACATCTAG
MvRNA_X_1562	1562	1591	29	ACTGGATTTCTCATTACCTTCATCTGCTA
MvRNA_X_1593	1593	1625	32	GATCAGGAATAATACCTATGAAGGCTGATCTT
MvRNA_X_1627	1627	1659	32	AACTTAAATGATGGTTGGACTGATACTGCACA
MvRNA_X_1675	1675	1707	32	TTGCTTGTTTTAGAGCTAATTGTATGGGCTGC
MvRNA_X_1738	1738	1770	32	TATCGAACATTATCTCTATTTAGGTACCGCAG
MvRNA_X_1776	1776	1808	32	AGAAGTCACTAACAATGTATGAGCCAATGCAG
MvRNA_X_1871	1871	1899	28	TATTGCCTTAATCCATCTGAGGCTACAC
MvRNA_X_2116	2116	2147	31	TAACATTTTGCTTTGGCTGGGTTCTGATACC
MvRNA_X_2149	2149	2180	31	GTCTTCATGGTTGGGCTACAGTATTGCTTTT
MvRNA_X_2182	2182	2210	28	ATTCTCTTGCTGTTGAGCTATGTGTGCC
MvRNA_X_2360	2360	2390	30	ACCGTGTTCAAAGATTCCGTGGTGCTGAAC
MvRNA_X_2392	2392	2422	30	TTTCAATATAAGCTCACCTACTTGCCTGAT
MvRNA_X_2457	2457	2489	32	AATGCACGGTTTTTTGTACTTTAGCAGGACCA
MvRNA_X_2594	2594	2623	29	AAAGGATGATCCTGTATATGTCTGGGCCC
MvRNA_X_2625	2625	2653	28	ACTCTATACATCTACTGGTGTGCTGACA
MvRNA_X_2655	2655	2687	32	ATACAGTACAAGGTATTGCATTTTCAGGCAGC
MvRNA_X_2763	2763	2792	29	CTTTTGCACCACATGGCGAAGATCATGAC
MvRNA_X_2794	2794	2824	30	TTATCGGTCAGCTGAGGTTCTGTCACGAAT
MvRNA_X_2826	2826	2853	27	GAGCCATTGTATGTTCCCACGCTGGAT
MvRNA_X_2855	2855	2887	32	AGGATATTACATCTGTCTTATTGGGAGTAGCT

MvRNA_X_2900	2900	2932	32	TGAAACTCTTGTTCAGAAGACTGGGTGTACGG
MvRNA_X_2941	2941	2973	32	AAGGTGACATCTGGCGACAGTATGAAGATTGA
MvRNA_X_2978	2978	3010	32	AGTGACCATAATGGTTCGCTGTTTTTTGGTCA
MvRNA_X_3014	3014	3040	26	CCTCTCTCAGCCAAGCCATACTTATG
MvRNA_X_3048	3048	3078	30	GTTGAAGGTGTCTGCTTCAACCCAATACAC
MvRNA_X_3119	3119	3151	32	TTGTCTTCTGGGTTTAGGAGATCAACGAATCC
MvRNA_X_3199	3199	3231	32	GACTTGTCATGTAATCAAACAGTATGTCAGCC
MvRNA_X_3284	3284	3316	32	TGAGGTCAATTTAAGAGGTCTGTGCTTGATCC
MvRNA_X_3412	3412	3444	32	TCTTGTAATTTTGATTTGCACACCAGTACAGC
MvRNA_X_3503	3503	3533	30	CTATTGGGCTAGGTCAAGGCCTTGTTGTAG
S-vRNA				
**name**	**start**	**end**	**size (nt)**	**sequence (3' → 5')**
SvRNA_Z_35	35	67	32	TATAAGTACACAATATACTACCTCAACATGCT
SvRNA_Z_152	152	184	32	TATTTAATGGGCCCAAACATGTGAAACTGAGC
SvRNA_Z_257	257	289	32	ATAACAATCAATACAGGGATCACTAATGGCAG
SvRNA_Z_496	496	528	32	AAGGTCAAGGAAATATCGAACCAGGAACCACT
SvRNA_Z_546	546	573	27	ATGGATCCAGAGCTCAGAGGTCTTGCA
SvRNA_Z_575	575	606	31	GAGATGGTAGATCACTTCCATCTTGGTGATG
SvRNA_Z_608	608	640	32	AATTATCCTCTTGTTTATGTTGGAATGGGGCA
SvRNA_Z_643	643	675	32	CGAACTCAATCAATGGGAATTCAGCTTGATCA
SvRNA_Z_677	677	709	32	GAAAAAGTCTTCCTTTTACCAATCTTACCTGC
SvRNA_Z_743	743	775	32	ATTGCAGGACATGAGAAACACAATAATGGCAT
SvRNA_Z_783	783	809	26	CAGGGATGGCAGAGCTTGGGGCCTTT
SvRNA_Z_811	811	838	27	CCGATGCCCACCAACATGTATCTATGT
SvRNA_Z_840	840	866	26	CACCATGGGTCTTTGCATGTGCACCA
SvRNA_Z_868	868	897	29	ACATCACCTGACAACATTGATTCGCCTAA
SvRNA_Z_899	899	928	29	GTTAATTGACTATGCAGCCTCTGGAGACC
SvRNA_Z_931	931	963	32	GTGCTTGACAAAAATCATGTGGCAGACATTGA
SvRNA_Z_965	965	997	32	AAGAAATAAGATCTACTTTATGCAGCGCCAGG
SvRNA_Z_1011	1011	1040	29	AAGTAAAACCAGGCACACCAGCACAGGAG
SvRNA_Z_1042	1042	1074	32	TTCATGGAAAAAGAGTGCCCATTCATAAAGCC
SvRNA_Z_1135	1135	1165	30	TCAGATCCAGGTTCGTAACATCATGAGTCC
SvRNA_Z_1167	1167	1199	32	GATTTCGCACAATAGTATGTGGTCTTTTTCCC
SvRNA_Z_1202	1202	1234	32	GTCAACTATGAAAGCAGAAGAACTCACACCTG
SvRNA_Z_1236	1236	1265	29	AGCATTTATATGTTTCTATGCCTACTGCC
SvRNA_Z_1267	1267	1299	32	TCATTTGAAGACATCAATGGCATAAGGAGACC
SvRNA_Z_1360	1360	1391	31	TCATCCTTAAAGCTTTATACATGCTCTCAAC
SvRNA_Z_1393	1393	1425	32	ATTGGAGTGTATGTGATAGGGTTCACACTTCC
SvRNA_Z_1445	1445	1477	32	AAATGCAATTGACATTGAAGAACCAAGTGGCC
SvRNA_Z_1510	1510	1540	30	GATTGAACCTGATGACCACCTCAAGGAGAG
SvRNA_Z_1542	1542	1574	32	AAAAAATGGATACTAAACCTACTGACCCGACT

SvRNA_Z_1592	1592	1624	32	GGACAAACTCGCAGACTACAAGAGAAGGATGG
SvRNA_Z_1626	1626	1653	27	GCCAGGCAACAAACAGTGTCAGCACTG
SvRNA_Z_1655	1655	1687	32	GGACCCAGATGACGTTAATAAAAACACACTGC
SvRNA_Z_1696	1696	1728	32	GCCAGACAAAAACTTAAGGATGCAGAGAGAGC
SvRNA_Z_1751	1751	1783	32	TGACTTGACAGATATCCAAGAGGATATAACCC
SvRNA_Z_1791	1791	1823	32	AGACTCCTTGAAAAGCTACTACGAGAAAGTCT
L-mRNA				
**name**	**start**	**end**	**size (nt)**	**sequence (3' → 5')**
PUUV_Lm_119_Z	119	146	27	TCATGCCTCACTGCATACAATCTATCC
PUUV_Lm_513_Z	513	545	32	GTTGATATGTTTGAACCATCAGTCCTAACTGC
PUUV_Lm_727_Z	727	759	32	TTCTAATATAGGTTGGCATAATGGCTGAGGTC
PUUV_Lm_2537_Z	2537	2566	29	CTAAATGACCATCTTCCTGTAATGCACCT
PUUV_Lm_2781_Z	2781	2812	31	ATGATATGTATTCCTCTGGGACAACTTTGGC
PUUV_Lm_2814_Z	2814	2845	31	GCTGAATATTAAGTATTTTCCTTTCACCCCC
PUUV_Lm_2848_Z	2848	2875	27	AGGCCCATCGAAGTGCCTTTTCAAGGG
PUUV_Lm_2877_Z	2877	2909	32	TGACCTAGTGAAGACTGTATTTCACTTTCACC
PUUV_Lm_3642_Z	3642	3670	28	ATCTGCTTTGAGCAGCTGCAAGATCATC
PUUV_Lm_3672_Z	3672	3700	28	GACATGCACCCATATCTAGTGCTTTGAC
PUUV_Lm_3702_Z	3702	3731	29	CATAGCACTATTCCTAACTGTGCAAGCTG
PUUV_Lm_3733_Z	3733	3764	31	GCAGTGCCATATAACCTTTCAACTTTACTTG
PUUV_Lm_4035_Z	4035	4067	32	TTAGGGGTAAACACTTTCCATTGAACTTTCCC
PUUV_Lm_4197_Z	4197	4228	31	TTGTCATTGCAGTAACTATGCTGGGATCATT
PUUV_Lm_4230_Z	4230	4262	32	TGCATTCTGAACCTTAATTGCAATGGTGATTG
PUUV_Lm_4565_Z	4565	4597	32	CATCACTTATCTCATCTACTTTACTTGCATAT
PUUV_Lm_4666_Z	4666	4698	32	ATTTGGTTGACTAATGTCAAGTCCCAATTCTC
PUUV_Lm_4749_Z	4749	4777	28	TACCCTCAACATTTCCTTGCACTATCAC
PUUV_Lm_4860_Z	4860	4892	32	TTCTCTTTCTTACCATAAAGTGATACAGCCTT
PUUV_Lm_4996_Z	4996	5026	30	CCCACTGCTCTAGTGTTTTATTCTCAAACA
PUUV_Lm_5169_Z	5169	5201	32	GTTTCCTCATTGTAAGTTTGCACACAATATGC
PUUV_Lm_5362_Z	5362	5394	32	AACTTGTTCTGTTTTAAATCTCATACCGCAGC
PUUV_Lm_5701_Z	5701	5733	32	ATTTTCTAGTAATCCAGCTGATATGATCGGGT
PUUV_Lm_5929_Z	5929	5961	32	ATCATCTAGTGTCAAACTTACTTCTTCTGACC
M-mRNA				
**name**	**start**	**end**	**size (nt)**	**sequence (3' → 5')**
PUUV_Mm_55_Z	55	87	32	AGACCCTGAAGAATCAGATACAGACAAACTGG
PUUV_Mm_133_Z	133	159	26	AGCCCAATAGTGTGCGGACACTCCAT
PUUV_Mm_161_Z	161	191	30	GTTCTACTGAACCTACAACAAGGCCTTGAC
PUUV_Mm_193_Z	193	225	32	AAGGATTCAATTTGCTGTATTGGGACAGGTGG
PUUV_Mm_369_Z	369	400	31	AGGGATCAAGCACAGACCTCTTAAATTGACC
PUUV_Mm_402_Z	402	429	27	ATCCTTGCTGCTGTTTCAACCACTAGG
PUUV_Mm_472_Z	472	504	32	GGTCCCATTAAATAAACAGTAGGCTGACATAC

PUUV_Mm_606_Z	606	638	32	TTGTGTGTATTGGGTTGAAGCAGACACCTTCA
PUUV_Mm_640_Z	640	670	30	ATCATAAGTATGGCTTGGCTGAGAGAGGGC
PUUV_Mm_824_Z	824	851	27	GCGTGGGAACATACAATGGCTCTGAGC
PUUV_Mm_853_Z	853	882	29	GACAGAACCTCAGCTGACCGATAATCATC
PUUV_Mm_948_Z	948	976	28	TGCTTTACCTGTAACCTTCCCAGCAATG
PUUV_Mm_1044_Z	1044	1070	26	GATCATCCTTTGCTGTCAGCACACCA
PUUV_Mm_1072_Z	1072	1102	30	CATAATAATTCCTGGGGCCCAGACATATAC
PUUV_Mm_1104_Z	1104	1136	32	GTGTCTTTTTTTCACAGACAGAGTGGTTTCCC
PUUV_Mm_1138_Z	1138	1167	29	AATGGGATGAACCCTGTCCATGTTAACGG
PUUV_Mm_1271_Z	1271	1303	32	TTGAACACGGTTTATCAGGCAAGTAGGTGAGC
PUUV_Mm_1471_Z	1471	1501	30	TGGCACACATAGCTCAACAGCAAGAGAATG
PUUV_Mm_1550_Z	1550	1582	32	GATCATTGTAATAGTTGGTATCAGAACCCAGC
PUUV_Mm_1693_Z	1693	1723	30	CTCATACTGACATACCTCACAAACCATTGA
PUUV_Mm_1760_Z	1760	1792	32	AAGGCAATAAGGGCATGAACCAATTGAACAGC
PUUV_Mm_1897_Z	1897	1929	32	AGAGATAATGTTCGATAACAACCCTGCATTGG
PUUV_Mm_2078_Z	2078	2110	32	AGGTAATGAGAAATCCAGTTCAAGATCAGCCT
PUUV_Mm_2227_Z	2227	2259	32	TTTAAGTTAAATGTAGCATCCATCCAGTGGCC
PUUV_Mm_2304_Z	2304	2335	31	TTTTTCAACGAAACATCCTGCTGTTTGCCAG
PUUV_Mm_2503_Z	2503	2535	32	TCATTACTGTCAACAGTTTTACAGGTTTGCCC
PUUV_Mm_2560_Z	2560	2592	32	TGGAACTTTGAAATGGTACCTATCAAGCACAC
PUUV_Mm_2613_Z	2613	2645	32	AAATTAAACCACCTTGTTGGAGAGGACCGAGG
PUUV_Mm_2649_Z	2649	2681	32	CACCAAATTGACATGTAGTAGTACACCATTGC
PUUV_Mm_2684_Z	2684	2712	28	CCTGTGGGTGTACTCATTATATCTCCGG
PUUV_Mm_2855_Z	2855	2886	31	TCCAATGCACTTGTAGAGATGTGTGGTTCTG
PUUV_Mm_2888_Z	2888	2918	30	GATCCCGAAGTGAACTATCTAGGTCAATCC
PUUV_Mm_3100_Z	3100	3132	32	TTCGTTGTAGTAGAACCATAACACATAGCAGC
PUUV_Mm_3140_Z	3140	3169	29	ACCAACAACATGAATTGTATTCTGCCCTC
PUUV_Mm_3204_Z	3204	3234	30	AACCCTGTACTAGAGCACTTTGTGTCATGA
PUUV_Mm_3236_Z	3236	3263	27	CACGATCCAAGTGTGGTGCAGCTGCAA
PUUV_Mm_3528_Z	3528	3560	32	TAATGGCATATAGGTAGTCATATGGTGACTGC
S-mRNA				
**name**	**start**	**end**	**size (nt)**	**sequence (3' → 5')**
PUUV_Sm_12_Y	12	44	32	ATTCCAGACTTTCTCGTAGTAGCTTTTCAAGG
PUUV_Sm_46_Y	46	78	32	GGTTATATCCTCTTGGATATCTGTCAAGTCAC
PUUV_Sm_80_Y	80	112	32	TTTGTCTGGCAACAATAAGTTGCTGTTCATGG
PUUV_Sm_115_Y	115	141	26	TTCCACTGCTCTCTCTGCATCCTTAA
PUUV_Sm_143_Y	143	175	32	GCAGTGTGTTTTTATTAACGTCATCTGGGTCC
PUUV_Sm_178_Y	178	205	27	CCAGTGCTGACACTGTTTGTTGCCTGG
PUUV_Sm_207_Y	207	236	29	ATCCTTCTCTTGTAGTCTGCGAGTTTGTC
PUUV_Sm_238_Y	238	267	29	ATCCATTTTTTTCCTGGACACAGCATCTG
PUUV_Sm_269_Y	269	300	31	AGGTTCAATCCCAGTCGGGTCAGTAGGTTTA

PUUV_Sm_302_Y	302	329	27	AGGCTTGATCTCTCCTTGAGGTGGTCA
PUUV_Sm_383_Y	383	413	30	ACTCCAATTGTATACCAATCTGCTGTTTGG
PUUV_Sm_415_Y	415	447	32	AAGGATGATAGGAAGTGTGAACCCTATCACAT
PUUV_Sm_460_Y	460	486	26	AGTCTGTCTCCCACGCGTTGAGAGCA
PUUV_Sm_593_Y	593	625	32	GTGTGAGTTCTTCTGCTTTCATAGTTGACTGG
PUUV_Sm_669_Y	669	700	31	TAACTGGACTCATGATGTTACGAACCTGGAT
PUUV_Sm_743_Y	743	773	30	CACTCTTTTTCCATGAACTCTCTGATTCTC
PUUV_Sm_775_Y	775	807	32	TGTGCCTGGTTTTACTTCAGGCTTTATGAATG
PUUV_Sm_809_Y	809	841	32	TATTTCTTTTTAACATCTCAATCTCCTGTGCT
PUUV_Sm_850_Y	850	876	26	GTCAAGCACATCCTGGCGCTGCATAA
PUUV_Sm_914_Y	914	941	27	GGTGATGTAGGGTCTCCAGAGGCTGCA
PUUV_Sm_943_Y	943	973	30	CCCATGGTGCATTAGGCGAATCAATGTTGT
PUUV_Sm_975_Y	975	1001	26	GGGCATCGGTCTGGTGCACATGCAAA
PUUV_Sm_1003_Y	1003	1031	28	GCCATCCCTGCAACATAGATACATGTTG
PUUV_Sm_1065_Y	1065	1097	32	ACAGTCTTAGATGCCATTATTGTGTTTCTCAT
PUUV_Sm_1099_Y	1099	1131	32	AGACTTTTTCTTTAATTTCTCTTCTGCTGTGC
PUUV_Sm_1167_Y	1167	1199	32	AGGATAATTCTTTGATCAAGCTGAATTCCCAT
PUUV_Sm_1218_Y	1218	1248	30	AAGATGGAAGTGATCTACCATCTCTTTGCC
PUUV_Sm_1251_Y	1251	1277	26	CCTCTGAGCTCTGGATCCATATCATC
PUUV_Sm_1279_Y	1279	1308	29	GACCTTTTGATCTATCAGTGCCTGTGCAA
PUUV_Sm_1310_Y	1310	1342	32	ATATCTTTAGTGGTTCCTGGTTCGATATTTCC
PUUV_Sm_1453_Y	1453	1485	32	ATTAATGACCCTGATAACTGCAACTATAAACC
PUUV_Sm_1656_Y	1656	1688	32	AAAAAGGACAGCTCAGTTTCACATGTTTGGGC

Table SM3: FISH-Probes used in experiments presented in Figure 7.

S-VRNA			
ATTCATTTCTACCTCAGTGT	GGCCCAAACATGTGAAACTG	GGGATCACTAATGGCAGTTA	TGCAGTTATCAGGGTCATTA
CAGGGATCATATGTGCTAGT	GTAGTGAGTTGTTAACATGT	TCCTTAATTACATATGCACT	ATATCGAACCAGGAACCACT
TTGCACAGGCACTGATAGAT	TATGGATCCAGAGCTCAGAG	ATCACTTCCATCTTGGTGAT	GAATGGGGCAAAGAGATGGT
AATTATCCTCTTGTTTATGT	ATGGGAATTCAGCTTGATCA	TTACCTGCGTCGAACTCAAT	TAAGACTGTTGGCACAGCAG
TCTTTGCATGTGCACCAGAC	GATTCGCCTAATGCACCATG	ACCCTACATCACCTGACAAC	TAATTGACTATGCAGCCTCT
TCATGTGGCAGACATTGACA	GCCAGGATGTGCTTGACAAA	AGTAAAACCAGGCACACCAG	GGAAAAAGAGTGCCCATTCA
TCATGAGTCCAGTTATGGGG	ACTCAGATCCAGGTTCGTAA C	ATTTATATGTTTCTATGCCT	TCAATGGCATAAGGAGACCA
AACGCGTGGGAGACAGACTG	CCTATCATCCTTAAAGCTTT	GTATGTGATAGGGTTCACAC	GCAGATTGGTATACAATTGG
ATTGAAGAACCAAGTGGCCA	TAAACCTACTGACCCGACTG	AGAGAAGGATGGCAGATGCT	CAAGCCAGGCAACAAACAGT
GGACCCAGATGACGTTAATA	GCAACTTATTGTTGCCAGAC	AGGATATAACCCGCCATGAA	AGTGACTTGACAGATATCCA
ACTGGAGGACAAACTCGCAG	AACTCACACCTGGCAGATTT	CCAGTCAACTATGAAAGCA	
M-VRNA			
TATTTGCATTATGTTGTCCA	AGCATTACTTATACTATCCA	TGGGAATTGGATGGTTGTGG	AAGATCTTTGATGATGGCGC
AAAGTGCTCTAGTACAGGGT	GGTAAAGGTGGACATTCTGG	GTGTTATGGTTCTACTACAA	AACATCAGCTACAGATGGGG

CCTGTATGTCAGTTTGATGG	AATTTGGTGATCCCGGAGAT	ACATCAGTTAAAGTGTGCTT	TTGCGGTGTATATCTGGATA
ATGAAACTGGTTGGGGTTGT	GTTGGACTGATACTGCACAT	CTGCTAGTGCTGAGACACAA	ATGTTGCTTGTTTTAGAGCT
TTTTGTAGGTTTAGTGTGGT	CTATTTAGGTACCGCAGTAG	GCAGGGTTGTTATCGAACAT	CTAACATCACGGTTTCAGGA
CTTATTGCCTTAATCCATCT	AGAGCTGTTCAATTGGTTCA	TGGTTTGTGAGGTATGTCAG	CATTTGCATATCTTTGCTCT
CTGGGTTCTGATACCAACTA	CACATTCTCTTGCTGTTGAG	ACCTTAGTTATTGGACAGTG	GTAGACATGGATATCACTGT
TTCTGAGACAGGTATTTTCA	AAGGTATTGCATTTTCAGGC	GGGAAGGTTACAGGTAAAGC	ACCACATGGCGAAGATCATG
TTCTGTCACGAATGGCTTTT	ACATCTGTCTTATTGGGAGT	CTCTTGTTCAGAAGACTGGG	GCCAAGCCATACTTATGATA
GGGACCTATTCAAACATGCT	ATGTCAGCCTACTGTTTATT	CCTAGTGGTTGAAACAGCAG	TAAGAGGTCTGTGCTTGATC
GCATCATCAACAAGTTTTCA	GGACATGGGAGACAAAAGGT	CTGTCCCAATACAGCAAATT	ACTTAAAATGGAGTGTCCGC
CTGGTATGTACTGTTAGTTT	ATGTGATTGTAAGCCGTGAT	TCACCTACTTGCCTGATAAA	CCACTCTATACATCTACTGG
L-VRNA			
AGGTATGTAAACTAGGTGGT	TAACCTTTTAGTGTACCTGG	CAGTATCACCTACAAATGCT	GCAGTTTAATATCAGAGGCA
TACCTACTCTGGCTTTGAAC	GGGTTTCTAAAGGTTTGTCT	GCAGTAGGAAATGTGTGGTT	TCAAGAAGTGTTGTGTTGTT
GTGGGTAGACTTATTTTGTT	GGTTGAAATACTTGGTGTTT	AGGAGGTATTAAAGGCTGTA	TGATGTATTGGATAGTGCCT
AGTTGCTCGTACATTCACAG	ATGGGTGACATTTAGAGAGG	TGAATGATCCCAGCATAGTT	TATCTAGAGCTTTGGAGTGA
GTGTTTACCCCTAAAAGTGA	CTGGGATAGGAATGGCAGAT	GGTTCTATGTCAATCATGGA	GTTTAACCTCCATGAACACA
GGTTTCAATATGTGACCCAA	GGAGCCTATAGATGATGGTA	TTTGGGGCAGCAATATCATT	GCAGATGCAACTAAGTGGTC
TCCTACAGTACTTGAACTTT	AGTCTTAAAGGTATGTCAGG	ATCCACCTAGAAACAGTTGA	AAACAACAGCCTGGCATCTA
GTAGCAAGTCAAGTACAGCA	AAGGAGTGTTCGGTGAGTTG	CCAAGGGTTTACTTTAAGCC	CTGAGACACCATTACAGCTA
CCATCAACAATTGCATCTGA	TATAGACCTCAGCCATTATG	TCGTACATTTGATATTCCGA	GGCGTTGTTCAAGCAATGAG
GTGGCCTAGTAGAAGGAATG	GTTACAGATAGAAGCTCAGA	GCAGGTGTTCCAAATGATGT	ACGATAGATACGTTCTGGGT
CGCAATCTTTGATAATCTCC	CCTAATGACTCGGTATATGT	TAAAGAGTTGTTGCGCTGCG	CTGAGGCTGATAGAGGTTTT
GGTTACTTTGATGATCTTGC	TATCGAGGTTTGGAGATGGT	TTTCTTCTTGCACATGCTTA	
L-mRNA			
tccttgactctctcatgaat	taggccactgtgttgatatg	cttgaacaacgccttcattc	aaccttaggacctgttacat
gctgaggtctataattcgga	atctgttccaagccaatttt	ggtgactgcaagctaatttg	acaccagttgttttcttagg
taaatgtcctagatgccagg	gacactaccacatgcataac	atgcaatattcaaggccagt	ttggtcaacagttaacccta
ccattcaactgtttctaggt	tgagcagctagttcgacaac	ttcataatgttgcttgccat	taatgcacctgacataccтt
gctgctaaatgaccatcttc	taacaattcttgcttgtgcc	gggacaactttggcaatagc	acttagttgcatctgcacta
ttgctgccccaaataatgaa	actctgggtacaatttagcc	gaaaccactcagtaccatca	ttgcctgcttgaatttgttg
ccatcaccacctaaaggaat	ctgttgctaactccatgatt	cccaacaaagctgaactctc	ggggtaaacactttccattg
tgcagtaactatgctgggat	taccttcatatgctgtttcg	atgtaggccgatattcagac	acatttccttgcactatcac
ctacagattgactcagctgt	agagacatagttttcagcca	acatagtctcattgcagcta	ctgggtcaataggcaatacc
cctcattgtaagtttgcaca	ttaaatctcataccgcagcg	agccactctctgtcttaaac	cctgttcggagagtacaatt
gaatgctttatttgtcgggt	ttttcttcatcaagctgtgc	cagctgatatgatcgggttt	cactgtgtgctagagattgt
atcagctttacaggctcaag	actgctttgcctggaaatta	ttcgttgagcttcttgatca	agaagaggctgaatccctag
M-mRNA			
ctgcaagatcaccttttgtc	gtgtattgggttgaagcaga	taagtatggcttggctgaga	gcgaaccattatggtcacta
aatcttcatactgtcgccag	cttgaaaaccattggccgta	gagctactcccaataagaca	atgtggtgcaaaagccattc
gatggtgctttacctgtaac	ggctgcctgaaaatgcaata	cagcacaccagtagatgtat	cccagacatatacaggatca
tgttaacggcagtgtctttt	tgcattgtgttgtcttttca	ggcaagtaggtgagcttata	agcaccacggaatctttgaa
gcaattcctggaatcatgga	tggcacacatagctcaacag	aagcaatactgtagcccaac	tacctcacaaaccattgagc
cttgaagagcagacggtgta	caagcaacatacaccacact	aagttttgtgtctcagcact	agtatcagtccaaccatcat

ggtattattcctgatccatg	tgtcttcggtaagtatagct	gtggcctagatgttgaattt	tcaacgaaacatcctgctgt
caggacaatcagggggatta	acccaactgaatgcatactt	atggtacctatcaagcacac	ttggagaggaccgaggaata
atgtagtagtacaccattgc	cattatatctccgggatcac	aagttctggacacttcatcc	gtattcccatcaaactgaca
ccatccgtttataacctgaa	taggtcaatccactccaatg	tgtgatcccgaagtgaacta	accaggttaaaaccaactcc
gcagagcattctgttaaact	attcgttgtagtagaaccat	acatgaattgtattctgccc	gtactagagcactttgtgtc
gattaaaccctgtcacacga	cagcaagacataccacactc	agtaatgctatcaacacagc	gatctttcttataactgggc
S-mRNA			
ataagttgctgttcatggcg	gcatccttaagtttttgtct	gcttgcagtgtgtttttatt	agtgctgacactgtttgttg
tcttgtagtctgcgagtttg	attgtataccaatctgctgt	gaagtgtgaaccctatcaca	acgcgttgagagcatgtata
gccattgatgtcttcaaatg	ataaatgctttggtctcctt	ctgggcagtaggcatagaaa	gttcttctgctttcatagtt
tgtgcgaaatctgccaggtg	tctttttccatgaactctct	ttcaggctttatgaatgggc	tggtgcattaggcgaatcaa
ctcatgtcctgcaatattga	tgtgccaacagtcttagatg	ccccattccaacataaacaa	gaagtgatctaccatctctt
gatccatatcatcaccaaga	ttttgatctatcagtgcctg	atatctttagtggttcctgg	

Confocal Microscopy. Confocal microscopy was conducted with the inverted laser scanning microscope Olympus FluoView FV-1000MPE (Olympus, Hamburg, Germany). Images were obtained with a 60×/1.45oil-immersion objective at 25°C with a resolution of 512 x 512 pixels applying sequential scanning. DAPI or Hoechst 33342 were excited with a diode laser at 405 nm and detected between 425 and 475 nm. FITC was excited at 488 nm with an argon laser and detected in the range of 500-600 nm. TRITC and TAMRA were both excited with a diode laser at 559 nm and detected in the range of 570 – 625 nm. Cy5 was excited with a diode laser at 635 nm and detection began at 650 nm. Images were analyzed with the latest version of Olympus FluoView (Olympus, Hamburg, Germany).

MuSeq-FISH was acquired on a Visitron VisiScope spinning-disk confocal (SDC) laser microscope (Visitron Systems, Puchheim, Germany), using a 60×/1.45 UplanSApo oil immersion objective. The microscope was equipped with a Yokogawa CSU-W1 scan head and an Andor iXon 888 EMCCD camera (1024 × 1024 pixels, Andor, Belfast, Northern Ireland). Fluorescence microscopy was carried out using the following diode lasers: 488 nm (ATTO 488/ AlexaFluor 488) with an ET525/50-nm emission filter, 561 nm (ATTO 550) with an ET600/50-nm emission filter,

640 nm (ATTO 647N/ AlexaFluor 647) with an ET700/75-nm emission filter and 405 nm (DAPI) with an ET460/50-nm emission filter. To avoid nonspecific fluorescence detection, sequential imaging was performed. Additionally, an incubator unit was used to avoid turbulence and temperature fluctuations in the microscope environment. If not otherwise mentioned images were acquired with an image depth size of 16-bit, a resolution of ~200 × 200 × 700 nm and with a 0.2-μm z-step size with 40–46 slices spanning the entire cell volume. VisiView® software (Visitron Systems, Puchheim, Germany) was used for controlling microscope automation and image acquisition. Prior to the automatic image acquisition via stage positions, one differential interference contrast (DIC) image was taken from the center of the 15 well ibidiTreat μ-slide (ibidi GmbH, Munich, Germany). This image served as template in further runs to align the stage position. Images were automatically recorded in a 4 × 4 grid pattern at the center of each well using XYZ-Stage and AutoFocus module.

Line scans. Line scan analysis was performed using Fiji/ImageJ. Linear regions of interest (ROIs) were manually drawn across selected subcellular areas of interest—such as vRNA- or N protein-positive puncta, actin fibers, or microtubule bundles—based on visual identification of signal enrichment. This approach was chosen to enable precise assessment of fluorescence intensity profiles across relevant local structures, rather than across the entirety of the cell, which could obscure discrete signal overlaps due to spatial heterogeneity. Normalized intensity profiles for each fluorescence channel were extracted along these ROIs and plotted using Prism (GraphPad) or R. Representative line scan regions are indicated as dashed lines in the corresponding microscopy images.

Image segmentation and analysis using CellProfiler. Briefly, our pipeline identifies nuclei based on DNA counterstaining, followed by recognition of cellular bodies in either IF stainings of

abundant cellular markers (such as microtubules or actin), or in transmission light images (differential interference or phase contrast). Then, P-Body, N protein or vRNA puncta were identified independently. Finally, identified objects were saved as regions of interest (ROI) and quantitatively assessed.

Multicolor 3D fluorescence microscopy data from conventional or spinning disc confocal microscopy were first transformed into maximum intensity projections using Fiji [7] and then processed using CellProfiler [8] version 4.2.5 using a customized pipeline. Briefly, the pipeline is composed of the following modules: 1) RescaleIntensity (adjustment of image intensity to improve segmentation), 2) CorrectIlluminationCalculate and CorrectIlluminationApply (correction of uneven illumination), 3) MorphologicalSkeleton (to improve cytoskeleton segmentation), 4) IdentifyPrimaryObjects (for identification of nuclei, vRNAs, mRNA, N protein, P-body spots, cytoskeleton), 5) IdentifySecondaryObjects (segmention of cells), 6) RelateObjects (assignment of spots to individual cells), 7) MeasureObjectIntensityDistribution (calculation of radial distributions), 8) MeasureColocalization (assessment of co-localization), 9) SaveImages and ExportToSpreadsheet for data export. Pipelines were adapted for specific experiments, adjusting for the availability of certain fluorescence channels, signal intensities, magnifications and cell densities. All pipelines are available upon reasonable request for validation and reproducibility purposes.

Radial distribution analysis. For radial intensity distribution analysis, the nucleus was segmented using DAPI or Hoechst signal and used as the origin for defining the cell center. The cytoplasm was then divided into 10 concentric radial bins (bin 1 = perinuclear, bin 10 = cell periphery) using CellProfiler's "MeasureObjectIntensityDistribution" module. Mean fractional intensity (MFrI) was computed for each bin and compared between mock and infected cells.

MuSeq-FISH. The principle behind this method (see also Figure S6) is that RNAs are detected with a first set of FISH probes, followed by a stringent formamide wash that removes all bound FISH probes (comparable to membrane stripping in immunohistochemistry). Subsequently, another round of FISH staining can be conducted, with a different set of FISH probes. Since washing and staining can be repeated multiple times, several nucleic acids can be detected and discriminated in the same fluorescence channels (see Materials and Methods for details). We used DAPI as a DNA counterstain and CellMask staining to facilitate subsequent, automated image segmentation and identification of cell bodies.

Image processing for MuSeq-FISH. Repeated cycles of image acquisition required the physical removal of the specimen from the microscope. This introduces offset that in the raw images, which were corrected in the *xy- and z*-planes using the 'Correct 3D-drift' and 'MultiStackReg' plugin in ImageJ [9–11].

3D Spot detection on the shift corrected images was conducted with FISH-quant [12] using a threshold with minimal hits for control samples, i.e., for non-infected cells. Briefly, FISH image z-stacks underwent filtering through the Laplacian of Gaussian (LoG) method, followed by spot detection using the local maximum approach. Cell bodies and their nuclei were identified by analyzing HSC CellMask deep red staining for the cytosol and DAPI staining for the nuclei using the built-in cell outline tool.

For co-localization analysis, spots were identified using a custom-written R script (R version 3.2.4, available via GIT-HUB (https://github.com/Budding-virus/Packbund) [13]. Briefly, the script: (i) loads all spot detection data frames of each image, (ii) scrambles the spot detection list of an image to avoid any bias, (iii) scores all spots within a cylinder (radius = 300 nm, height = 600 nm) into a single multi-component complex (MCC) as co-localizing by calculating

the Euclidian distance between individual spots, (iv) marks MCC assigned spots as used (excluded from further cluster analysis), (v) computes the center of mass for each MCC (starting point for a second colocalization analysis), and (vi) discards all MCCs with multiple copies of segments within one MCC. Spots in the nuclei were excluded from the co-localization analysis.

Spot detection and localization was also done on a simulated data set with a randomized distribution. This data set was generated by using a custom-written R script. For this purpose, different spot densities for each vRNA segment as well as Pb and N were distributed in simplified cell shapes (flat cuboid for the entire cell) by using the 'white noise' method. White-noise images are images where all pixels are drawn with equal intensities, and the signal power is independently distributed over time or among frequencies. Moreover, white noise is the mean-square derivative of the Wiener process or Brownian motion, and therefore represents the random distribution of elements in a three-dimensional space. Two different assumptions were made for the spot distribution per segment: (i) all segments have the same probability to occur in a cell and (ii) the segments have a different probability to occur in a cell. The probability chosen for the weighted occurrence of each segment was based on the average occurrence of each segment per cell from the FISH data.

Strand specific qRT-PCR. Infected cells were lysed in 350 µL RLT Lysis Buffer from the RNeasy Mini Kit (Qiagen GmbH, Hilden, Germany). The supernatant (140 µL) was mixed with 560 µL AVL Lysis Buffer from the QIAamp Viral RNA Mini Kit (Qiagen GmbH, Hilden, Germany). Both extractions were processed according to manufacturer's instructions. RNA from cells and supernatant was diluted in 80 µL of RNase-free water and AVL buffer, respectively. To ensure specific amplification of either messenger (m) or viral (v) RNA during cDNA synthesis we developed stand-specific primers that included an anchor sequence attached to their 5' end. A

primer targeting this anchor sequence was subsequently used for PCR amplification together with the corresponding gene-specific primer. Primer sequences and primer probe combinations are given in Table SM4 and Table SM5.

For cDNA synthesis, the Super-Script III reverse-transcription (Invitrogen, ThermoFisher Scientific, Waltham, MA, USA) was used. A mix of 2 µL strand-specific primer (10µM), 0.4 µL dNTP (25mM each), 7.4 µL RNase-free water and 5 µL RNA was incubated at 65 °C for 5 minutes, followed by a 1 minute incubation at 4°C. A second mix of 4 µL 5xPuffer, 1 µL DTT, 1 µL RiboLock RNase inhibitor (Invitrogen) and 1 µL SSIII-RT was added and the mix was incubated at 55 °C for 35 minutes before cooling down to 4°C again.

For PCR amplification the FastStart Essential DNA Probe Master (Roche, Basel, Switzerland) was used according to manufacturer's instructions in 20 µL total volume, using 10 µL Master Mix and 0.4 µL of each primer and probe (10 µM each) and 5 µL cDNA template. Cycling conditions were 10 minutes 95 °C followed by 45 cycles of 10 seconds 95°C and 30 seconds at 60 °C and a final cool down to 4°C.

Table SM4: Sequences of primer and probes used in experiments presented in Figure S8 (Supplementary Material). Bold nucleotides represent anchor sequence.

**Reverse Transcription (RT) primers**		
**name**	**strand**	**sequence (5' → 3')**<br>**()**
PUUV-Sot_S_F_anch	vRNA	**TCACCTTGGCGAGTAGC**GAAGTGGACCCAGATGACGTTAA
PUUV-Sot_S_R_anch	mRNA	**TCACCTTGGCGAGTAGC**CCKggACACAYCATCTgCCAT
PUUV-Sot_M_F_anch	vRNA	**TCACCTTGGCGAGTAGC**AGTTGGGTACRGAGCAAACCTG
PUUV-Sot_M_R_anch	mRNA	**TCACCTTGGCGAGTAGC**ATTAAACCACCYTGTTGGAGAG
PUUV-Sot_L_F_anch	vRNA	**TCACCTTGGCGAGTAGC**TGAACAGCACCCAGTTTATGACTA
PUUV-Sot_L_R_anch	mRNA	**TCACCTTGGCGAGTAGC**GGTGATTGCATTGTCATTGCAG
anchor		TCACCTTGGCGAGTAGC
**Gene-specific PCR primers**		
**name**	**segment**	**sequence (5' → 3')**
PUUV F	S	gARRTggACCCRgATgACgTTAA
PUUV R	S	CCKggACACAYCATCTgCCAT
PUUV_M F_v2	M	AGTTGGGTACCGAGCAAACCTG

PUUV_M R_v2	M	ATTAAACCACCTTGTTGGAGAGGAC
PUUV-Sot_L_F	L	TGAACAGCACCCAGTTTATGACTA
PUUV_L R	L	GGTGATTGCATTGTCATTGCAG
**qPCR probes**		
**name**	**segment**	**sequence (5' → 3')**
PUUV TMGB3	S	FAM-CAACARACAgTgTCAgCA-NFQ-MGB
M+_FAM	M	FAM-CTACATCAGTTAAAGTGTGCTTGATAGGT-BBQ-MGB
L+_FAM	L	FAM-ACCTGGTAAGAAAATTGAATGATCCCA-BBQ-MGB

Table SM5: Primes and probes probe combinations used in experiments presented in Figure S8 (Supplementary Material).

**Primer combinations used**					
**targeted strand**	**segment**	**RT primer**	**PCR primer F**	**PCR primer R**	**PCR probe**
vRNA	S	PUUV-Sot_S_F_anch	anchor	PUUV R	PUUV TMGB3
mRNA	S	PUUV-Sot_S_R_anch	PUUV F	anchor	PUUV TMGB3
vRNA	M	PUUV-Sot_M_F_anch	anchor	PUUV_M R_v2	M+_FAM
mRNA	M	PUUV-Sot_M_R_anch	PUUV_M F_v2	anchor	M+_FAM
vRNA	L	PUUV-Sot_L_F_anch	anchor	PUUV_L R	L+_FAM
mRNA	L	PUUV-Sot_L_R_anch	PUUV-Sot_L_F	anchor	L+_FAM

Infection of human pulmonary microvascular endothelial cells. For the infection of primary endothelial cells, human pulmonary microvascular endothelial cells (HPMEC, Promocell, Germany) were grown in Endothelial Cell Growth Medium MV (Promocell) supplemented with 100 U/ml penicillin and 100 µg/ml streptomycin (PAA Laboratories GmbH, Austria) under standard conditions. For the infection experiment, HPMEC were seeded at $6x10^4$ cells per well in a µ-Slide 8 Well ^{high} (Ibidi GmbH, Germany) and cultivated 48 h before infection. PUUV infection (strain Kazan) was performed as previously described[14] with a MOI of 0.5. Fixation, staining and microscopy was performed at 72 hpi if not otherwise stated. Briefly, the cells were rinsed twice with PBS and subsequently fixed in 3.7 % formaldehyde for 30 min.

For immunofluorescence staining of PUUV-infected HPMECs, fixed cells were washed twice in PBS and subsequently incubated with 2 % BSA (Miltenyi Biotec, Germany) for 30 min. The cells were permeabilized in 0.1 % Triton-X (Sigma-Aldrich), washed twice and stained with

an anti-Hantavirus nucleocapsid protein antibody (1:200, abcam, ab34757) and anti-Dcp1a for P-body detection (1:200, Abcam, ab183709). Filamentous actin was stained with Phalloidin-iFluor 555 Reagent (Abcam). Subsequently, the cells were incubated with secondary antibodies, conjugated with different AlexaFluor 488 or 647 (1:1000, Cell Signaling Technologies). All antibody incubations were carried out in BSA-containing PBS for 1 h at 37°C. Finally, the cells were washed again three times and analyzed using a Leica Stellaris 8 laser scanning confocal microscope (CLSM).

Images of HPMECs were acquired on a Leica Stellaris 8 laser scanning confocal microscope with LASX 4.7.0.28176 equipped with a 405 nm diode laser and a Leica White Light Laser (WLL) using a Leica HC PL APO CS2 20x/0.75 DRY objective lens. The pinhole diameter was set to 1 AU. The acquisition parameters were: for DAPI excitation with 405 nm diode laser at 3.61 %, detection at 430 nm – 621 nm with a HyD S-detector; for Alexa 488 excitation with 498 nm laser at 2.43 %, detection at 430 nm – 621 nm with a HyD S-detector; for Alexa 649 excitation with 651 nm laser at 6.83 %, detection at 661 nm – 750 nm with a HyD X 4-detector; for Phalloidin excitation with 553 nm laser at 2.85 %, detection at 558 nm – 737 nm with a HyD S 3-detector. Pixel size was 0.109 µm in x and y. The zoom was set to 1x, images were acquired in frame-sequential mode with a scan speed of 400 Hz (unidirectional), the pixel dwell time for all channels was 0.2625 µs.

Creation of hierarchical assembly trees (example). As an example, an MCC composed of the components Cx, Cy, Cz could have formed in three ways: 1) Cx joined a MCC composed of Cy and Cz, 2) Cy joined a MCC consisting of Cx and Cz, or 3) Cz joined the MCC composed of Cx and Cy. The most likely assembly order is then estimated by taking into account the abundance of

Cx, Cy, Cz and the respective binary complexes (*k*-1). The complete assembly tree for larger complexes is formed iteratively.

SUPPLEMENTARY METHODS BIBLIOGRAPHY

1. ‘*Puumala virus genome*’, *GenomeNet*
2. ‘*Stothard, P. PCR Primer Stats. (2004).*’,
3. Lu & Mathews. ‘*OligoWalk: an online siRNA design tool utilizing hybridization thermodynamics*’, *Nucleic Acids Res.* (2008).10.1093/nar/gkn250
4. Zuker. ‘*Mfold web server for nucleic acid folding and hybridization prediction*’, *Nucleic Acids Res.* (2003).10.1093/nar/gkg595
5. Zadeh, Steenberg, Bois, Wolfe, Pierce, Khan, Dirks & Pierce. ‘*NUPACK: Analysis and design of nucleic acid systems*’, *J. Comput. Chem.* (2011).10.1002/jcc.21596
6. ‘*NCBI. BLAST: Basic Local Alignment Search Tool*’,
7. Schindelin, Arganda-Carreras, Frise, Kaynig, Longair, Pietzsch, Preibisch, Rueden, Saalfeld, Schmid, Tinevez, White, Hartenstein, Eliceiri, Tomancak & Cardona. ‘*Fiji: an open-source platform for biological-image analysis*’, *Nat. Methods* (2012).10.1038/nmeth.2019
8. Carpenter, Jones, Lamprecht, Clarke, Kang, Friman, Guertin, Chang, Lindquist, Moffat, Golland & Sabatini. ‘*CellProfiler: image analysis software for identifying and quantifying cell phenotypes.*’, *Genome Biol.* (2006).10.1186/gb-2006-7-10-r100
9. Thevenaz, Ruttimann & Unser. ‘*A pyramid approach to subpixel registration based on intensity*’, *IEEE Trans. Image Process.* (1998).10.1109/83.650848
10. Parslow, Cardona & Bryson-Richardson. ‘*Sample Drift Correction Following 4D Confocal Time-lapse Imaging*’, *J. Vis. Exp.* (2014).10.3791/51086
11. Tseng, Wang, Duchemin-Pelletier, Azioune, Carpi, Gao, Filhol, Piel, Théry & Balland. ‘*A new micropatterning method of soft substrates reveals that different tumorigenic signals*

can promote or reduce cell contraction levels', *Lab Chip* (2011).10.1039/c0lc00641f

12. Mueller, Senecal, Tantale, Marie-Nelly, Ly, Collin, Basyuk, Bertrand, Darzacq & Zimmer. '*FISH-quant: automatic counting of transcripts in 3D FISH images*', *Nat. Methods* (2013).10.1038/nmeth.2406

13. Haralampiev, Prisner, Nitzan, Schade, Jolmes, Schreiber, Loidolt-Krüger, Jongen, Chamiolo, Nilson, Winter, Friedman, Seitz, Wolff & Herrmann. '*Selective flexible packaging pathways of the segmented genome of influenza A virus*', *Nat. Commun.* (2020).10.1038/s41467-020-18108-1

14. Bourquain, Bodenstein, Schürer & Schaade. '*Puumala and Tula Virus Differ in Replication Kinetics and Innate Immune Stimulation in Human Endothelial Cells and Macrophages*', *Viruses* (2019).10.3390/v11090855
